# Effectiveness of combining PCSK9 inhibitors with statins on major adverse cardiovascular events and lipid levels in patients after percutaneous coronary intervention: a systematic review and meta-analysis

**DOI:** 10.3389/fcvm.2025.1612095

**Published:** 2025-10-29

**Authors:** Zhantao Cao, Ningjing Chen, Jun Chen, Xuejing Xu, Zhanglu Zhang, Yunsu Wang

**Affiliations:** Department of Cardiology, Xiamen TCM Hospital Affiliated to Fujian University of Traditional Chinese Medicine, Xiamen, Fujian, China

**Keywords:** PCSK9 inhibitors, statin (HMG-CoA reductase inhibitor), post PCI, major adverse cardiovascular events (MACE), LDL-cholesterol, lipid, meta -analysis

## Abstract

**Background:**

Few percutaneous coronary intervention (PCI) patients achieve low-density lipoprotein cholesterol (LDL-C) targets with statins alone. While proprotein convertase subtilisin/kexin type 9 (PCSK9) inhibitors effectively diminish LDL-C levels, their combined use with statins for reducing major adverse cardiovascular events (MACE) and improving lipid profiles post-PCI requires further validation. This study seeks to appraise the therapeutic impact of PCSK9 inhibitors combined with statins on MACE and blood lipids in patients following PCI.

**Methods:**

Randomized controlled trials (RCTs) and cohort studies as of February 2025 in the PubMed, Embase, Cochrane Library, and Web of Science databases were identified. Regarding the risk of bias evaluation, Cochrane ROB 2.0 was employed for RCTs. Moreover, cohort studies were appraised by means of the Newcastle-Ottawa Scale. In terms of heterogeneity, it was appraised by means of the *I*^2^ statistics. The relative risk (RR) and 95% confidence interval (CI) for dichotomous variables, along with the weighted mean difference (WMD), standardized mean difference (SMD), and their respective 95% CIs for continuous variables.

**Results:**

The meta-analysis included 17 studies, including 9 RCTs and 8 cohort studies, involving 5,607 subjects. The meta-analysis revealed that, against the statin group, the combination therapy group displayed a notable decline in MACE incidence (RR: 0.61; 95% CI: 0.50–0.75; *p* < 0.001; *I*^2^ = 0.0%). Meanwhile, the combination therapy group demonstrated greater LDL-C reduction vs. statin monotherapy (SMD: −1.29; 95% CI: −1.70 to −0.87). Moreover, The combination therapy group achieved significantly higher LDL-C ≤ 1.4 mmol/L attainment rates vs. statin monotherapy (RR: 5.83; 95% CI: 5.20–6.55).

**Conclusion:**

PCSK9 inhibitors combined with statins significantly reduces MACE incidence, improves lipid profiles in post-PCI patients compared to statin monotherapy.

**Systematic Review Registration:**

https://www.crd.york.ac.uk/, identifier (CRD420250650716).

## 1 Introduction

Cardiovascular diseases (CVDs) remain the leading cause of death globally, with a dominant contribution from atherosclerotic CVD (ASCVD) (1). Percutaneous coronary intervention (PCI) is a key method for revascularization in ASCVD patients, noticeably improving their prognosis. With the continuous advancement of PCI in recent years, its indications have become increasingly diverse. However, patients still face a pronounced residual risk postoperatively (2). Research indicates that plaque vulnerability and other risk factors contribute to a 15%–20% rate of major adverse cardiovascular events (MACE) within one year following PCI (3, 4). The pathological mechanism of atherosclerosis is closely tied to the abnormal deposition of low-density lipoprotein cholesterol (LDL-C) beneath the vascular endothelium. This lipid particle can provoke a chronic inflammatory response in the vessel wall, eventually causing plaque formation (5). Moreover, the marked elevation of LDL-C levels is highly connected to the occurrence and progression of ASCVD (6).

Statins are the cornerstone of lipid-lowering therapy, noticeably diminishing LDL-C levels by blockading HMG-CoA reductase. For patients following PCI, several guidelines suggest high-intensity statin therapy to reach a target LDL-C level of ≤1.4 mmol/L and a ≥50% decline from baseline (7, 8). However, even with intensive statin therapy, postoperative patients often exhibit low compliance rates in maintaining LDL-C levels (9). Furthermore, the effectiveness of this treatment in preventing myocardial infarction (MI) or cardiovascular mortality (CVM) is limited (10).

Inhibitors of proprotein convertase subtilisin/kexin type 9 (PCSK9) notably decrease plasma LDL-C levels by preventing the binding of PCSK9 protein to LDL-C receptors (LDLR) on hepatocyte surfaces, thereby decreasing LDLR degradation. In 2019, guidelines for managing dyslipidemias from the ESC/EAS emphasize that PCSK9 inhibitors should be added for patients with insufficiently controlled LDL-C levels to achieve the target levels (8). Combining PCSK9 inhibitors with statins has been proven to lead to a 60%–70% reduction in LDL-C levels (11). Additionally, multiple studies have demonstrated that PCSK9 inhibitors can noticeably diminish MACE risks in ASCVD patients (12–15). Key trials investigating the efficacy of PCSK9 inhibitors, like ODYSSEY, primarily include patients with acute coronary syndrome (ACS) (15). Nonetheless, post-PCI patients may have a higher risk of MACE compared to those who have not undergone surgery, due to factors like plaque vulnerability. The existing studies of patients after PCI have varied in the incidence of MACE and the degree of lipid improvement. Thus, we execute a systematic review and meta-analysis to appraise the overall effect of PCSK9 inhibitors in combination with statins on MACE and lipid levels for post-PCI patients, and to compare the degree of risk reduction with statin monotherapy.

## 2 Methods

This study complied with the Preferred Reporting Items for Systematic Reviews and Meta-Analyses (PRISMA) guidelines (16).

### 2.1 Data sources and retrieval strategies

PubMed, EMBASE, Cochrane Library, and Web of Science databases were searched for randomized controlled trials (RCTs) and cohort studies up to February 2025. Only English articles were considered. Both subject words and free words were incorporated in the retrieval method, including: “Percutaneous Coronary Intervention,” “PCSK9 Inhibitors,” “Evolocumab,” “Alirocumab,” and “Statins.” Two investigators (C.Z. and C.N.) independently searched relevant studies. Disagreements were settled by consulting a third investigator (W.Y.) to decide the final search results. Detailed retrieval strategies are provided in the Supplementary Table 1.

### 2.2 Inclusion and exclusion criteria

Studies satisfying the following criteria were incorporated: (i) population: Patients undergoing PCI (≥18 years old); (ii) interventions and comparisons: The interventions involved PCSK9 inhibitors (evolocumab, alirocumab) combined with statins, while the control group received either statin monotherapy or a placebo, with no restrictions on the dosages of various medications; (iii) outcomes: The outcome assessed was the incidence of MACE and lipid profile indicators. As the definition of MACE can vary across studies, data for the composite endpoint were extracted as defined by each individual study. For the purposes of this analysis, MACE was defined as a composite of cardiovascular mortality, non-fatal myocardial infarction, and non-fatal stroke, with some studies also including rehospitalization for unstable angina or unplanned revascularization in their composite endpoint. Data for each of these individual components were also extracted and analyzed separately where available; (iv) study designs: RCTs and cohort studies; (v) english studies. Studies were removed due to (i) subjects that did not undergo PCI or were <18 years old; (ii) outcomes that were not reported to be linked to MACE, or lipid levels; (iii) animal studies, conference materials, case reports, letters, reviews, proposals, and meta-analyses; (iv) unavailable full text. According to the aforementioned criteria, two investigators (C.Z. and C.N.) independently selected studies. If discrepancies occurred, they were addressed through consultation with a third investigator (W.Y.).

### 2.3 Data extraction

Two investigators (C.Z. and C.N.) independently extracted all relevant data from the incorporated studies, including the first author, publication year, country, study design, sample size, and treatment duration. Additionally, they collected baseline characteristics of patients in each study, which included age, sex, body mass index, clinical classification (ACS or acute MI [AMI] or ASCVD), types of PCSK9 inhibitors employed, and types of statins administered. The outcomes assessed included the incidence of MACE and baseline lipid values as well as achieved values.

### 2.4 Risk of bias (ROB) assessment

We leveraged the Cochrane Risk of Bias Tool 2.0 (ROB 2.0) to appraise the ROB in RCTs (17), including biases from the randomization process, deviations from intended interventions, missing outcome data, measurement of outcomes, selection of reported results, and overall bias. The ROB of each RCT was classified as low risk, some concerns, or high risk.

For cohort studies, the Newcastle-Ottawa Scale (NOS) was employed. The scores ranged from 0 to 9. If the score ≥7, a study would be of high quality (18). The NOS consists of eight items, categorized into three dimensions: selection, comparability, and outcome.

Two investigators (C.Z. and C.N.) independently appraised the ROB in studies. Discrepancies were resolved by consensus with a third investigator (W.Y.).

### 2.5 Study selection

All statistical analyses were implemented utilizing the meta program in the Stata 15.0 software. Binary variables were reported utilizing relative risk (RR) and 95% confidence interval (CI), while continuous variables were expressed with weighted mean difference (WMD), standardized mean difference (SMD), and their 95% CI. In terms of heterogeneity, it was appraised through the *I*^2^ statistics. If *I*^2^ < 50%, suggesting low heterogeneity, a fixed-effects model was leveraged, or a random-effects model was chosen. A noticeable difference was defined as *P* < 0.05. Sensitivity analyses were also executed utilizing the leave-one-out method, to identify the sources of heterogeneity and appraise the robustness of the meta-analysis results. For outcomes mentioned in 10 or more studies, publication bias was appraised by means of Egger's test, Begg's test, visual inspection on funnel plots, and the imputation method. Finally, the quality of evidence was appraised utilizing the GRADE (Grading of Recommendations Assessment, Development and Evaluation) method (19). According to the GRADE framework, evidence quality was appraised across five domains: ROB, inconsistency, imprecision, indirectness, and publication bias. The quality was rated as high, moderate, low, or very low. Two investigators (C.Z. and C.N.) independently conducted the assessments. Discrepancies were resolved by consensus with an additional investigator (W.Y.).

## 3 Results

### 3.1 Study selection

**Figure 1:**
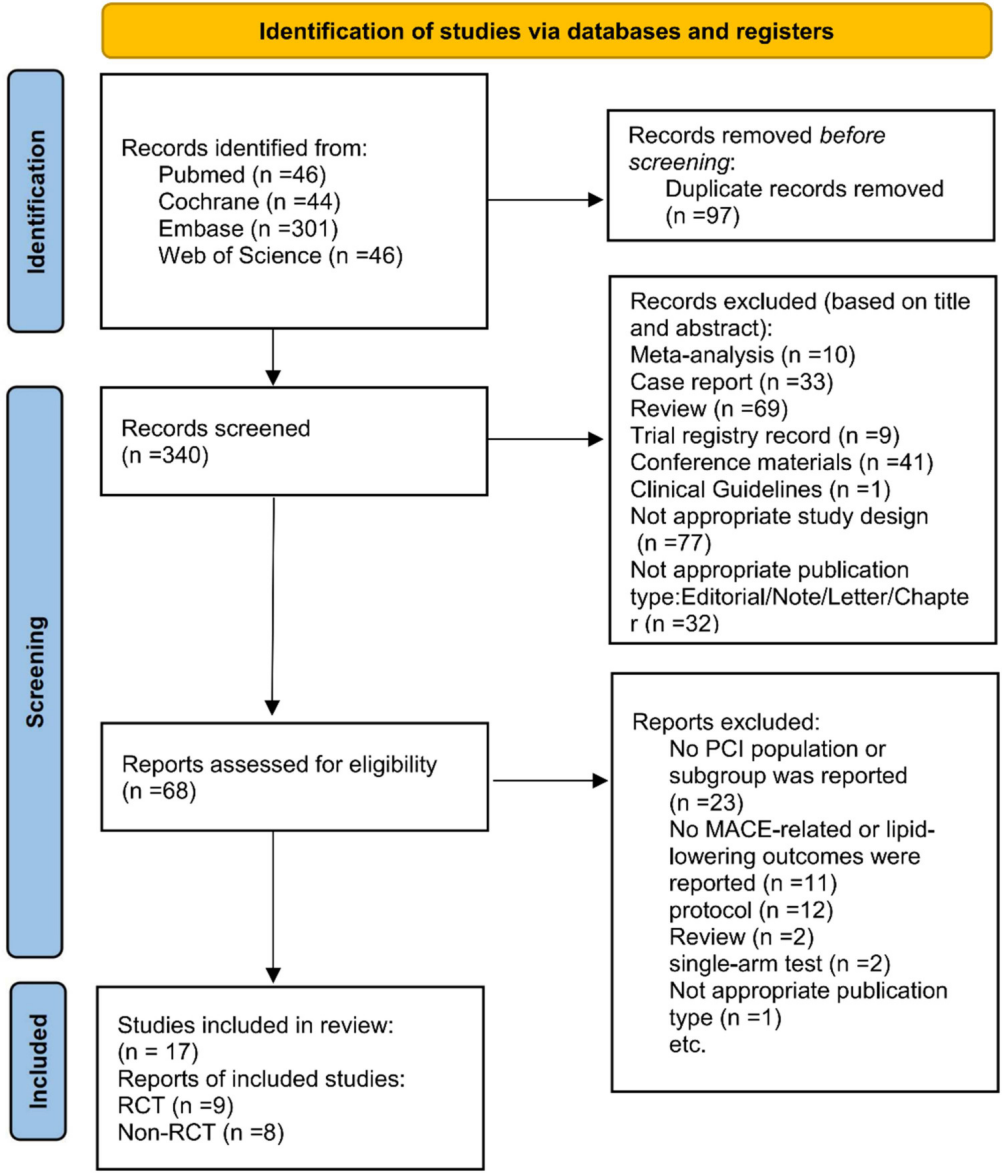
PRISMA flowchart of study selection.

437 related articles were initially retrieved using the above search methods: PubMed (*n* = 46), Embase (*n* = 301), Cochrane (*n* = 44), and Web of Science (*n* = 46). In these results, 97 duplicates were deleted. By reviewing the titles and abstracts, 272 irrelevant articles were eliminated. Subsequently, a full-text review was conducted on the remaining 68 papers. Among these, 51 studies were removed due to: no reported PCI population or subgroup (*n* = 23), absence of MACE-related, lipid-lowering (*n* = 11), protocols (*n* = 12), reviews (*n* = 2), single-arm trials (*n* = 2), and inappropriate publication types (*n* = 1). In the end, 17 articles were included, encompassing 9 RCTs (20–28) and 8 cohort studies (29–36). Figure 1 illustrates the screening methodology, along with the number of studies included/excluded, along with the reasons behind exclusions.

### 3.2 Study characteristics

In total, 17 articles were included, encompassing 9 RCTs (20–28) and 8 cohort studies (29–36). 5,607 subjects were involved. These articles were published between 2020 and 2025. The proportion of male participants varied between 51.6% and 96.7%. Sample sizes varied considerably, from 52 to 1,564 participants. The average age was between 48.3 years and 66.9 years. In the included studies, 13 (22–25, 27, 28, 30–36) utilized evolocumab exclusively, 2 (21, 26) only employed alirocumab, and 2 (20, 29) involved evolocumab or alirocumab. The included studies utilized two different types of PCSK9 inhibitors. Treatment regimens included evolocumab 140 mg Q2W, 420 mg QM, 140 mg single dose and alirocumab 75 mg Q2W, 150 mg Q2W. The primary fundamental characteristics for all included articles are displayed in Table 1.

**Table 1:** Basic characteristics of included studies.

Study	Country	Sample size		Treatment duration	Age (years old)		Male *n* (%)		BMI		Participants	PCSK9 inhibitor	Outcomes	Study design
		T	C		T	C	T	C	T	C				
Hao	China	68	68	12 weeks	62.21 ± 12.31	62.22 ± 11.44	48 (70.59)	45 (66.18)	26.28 ± 3.84	25.70 ± 3.16	ACS	①	(a) + (b) + (c)	RCT
Ji	China	55	55	24 weeks	60.45 ± 11.67	61.23 ± 10.34	38 (69.1)	36 (65.4)	24.93 ± 1.68	25.31 ± 1.74	NSTE-ACS	①	(a) + (b) + (c) + (d)	RCT
Mehta	Canada	38	30	6 weeks	61.37 ± 11.04	63.63 ± 10.38	27 (71.05)	28 (93.33)	/	/	STEMI	②	(a) + (c)	RCT
Okada	Japan	52	50	4 weeks	66.4 ± 13.1	63.4 ± 14.0	43 (82)	47 (94)	24.4 ± 4.4	25.2 ± 4.5	AMI	①	(b)	RCT
Shi	China	104	104	24 weeks	62.57 ± 10.37	61.78 ± 11.74	58 (55.8)	55 (52.9)	/	/	ACS	①	(a) + (d)	RCT
Uehara	Japan	29	23	36 weeks	59.6 ± 11.9	60.0 ± 12.9	22 (75.8)	16 (69.5)	/	/	STEMI	③	(b) + (c) + (e)	RCT
Wang	China	35	30	4 weeks	48.34 ± 12.2	44.67 ± 12.51	30 (85.7)	29 (96.7)	26 ± 3.1	26 ± 2.9	STEMI	④	(a)	RCT
Yamashita	Japan	62	62	52 weeks	66.9 ± 10.2	66.0 ± 11.6	48 (77)	48 (77)	24.2 ± 3.9	24.0 ± 3.6	ACS	① or ③ or ⑤	(a) + (b) + (c)	RCT
Räber	Europe	148	152	52 weeks	58.4 ± 10.0	58.6 ± 9.4	124 (83.8)	119 (78.3)	27.3 ± 4.1	28.2 ± 4.5	AMI	②	(c) + (e)	RCT
Kim	Koreon	45	50	8 weeks	57.9 ± 11.2	65.3 ± 14.1	45 (90.0)	35 (77.8)	25.8 ± 3.4	24.1 ± 3.0	AMI	④	(a) + (b) + (c)	NRCT
Li	China	153	137	2 weeks	57.69 ± 7.69	57.31 ± 8.95	79 (51.6)	72 (52.6)	/	/	ASCVD	③	(b) + (c)	NRCT
Liu	China	434	434	24 weeks	60 ± 11.9	60 ± 8.93	294 (67.7)	287 (66.1)	24.88 ± 2.34	25.03 ± 2.83	ASCVD	① or ③	(a) + (b)	NRCT
Jin	China	161	160	104 weeks	59.14 ± 13.57	61.30 ± 11.35	127 (78.9)	136 (85)	22.51 ± 2.38	23.02 ± 2.29	ACS	①	(a) + (b)	NRCT
Yano	Japan	18	40	12 weeks.	64.6 ± 5.3	65.2 ± 6.2	14 (77.8）	31 (77.5)	24.4 ± 4.3	24.1 ± 5.5	ACS	①	(b) + (e)	NRCT
Zhang	China	313	313	78 weeks	61.9 ± 10.6	61.9 ± 10.0	196 (62.6)	183 (58.5)	/	/	ACS	①	(a) + (b) + (c)	NRCT
Yao	China	310	310	52 weeks	62.43 ± 11.86	62.26 ± 11.43	257 (82.9)	265 (82.6)	25.32 ± 3.32	25.39 ± 3.48	ACS	① or ⑤	(a)	NRCT
Zhang	China	414	1,150	78 weeks	62.1 ± 10.9	62.2 ± 10.0	258 (62.3)	681 (59.2)	/	/	ACS	①	(a) + (b) + (c)	NRCT

① Evolocumab 140 mg Q2W, ② Alirocumab 150 mg Q2W, ③ Evolocumab 420 mg QM, ④ Evolocumab 140 mg single dose, ⑤ Alirocumab 75 mg Q2W. (a) MACEs, (b) lipid levels, (c) adverse reactions, (d) inflammatory markers, (e) coronary plaque.

### 3.3 ROB assessment

**Figure 2:**
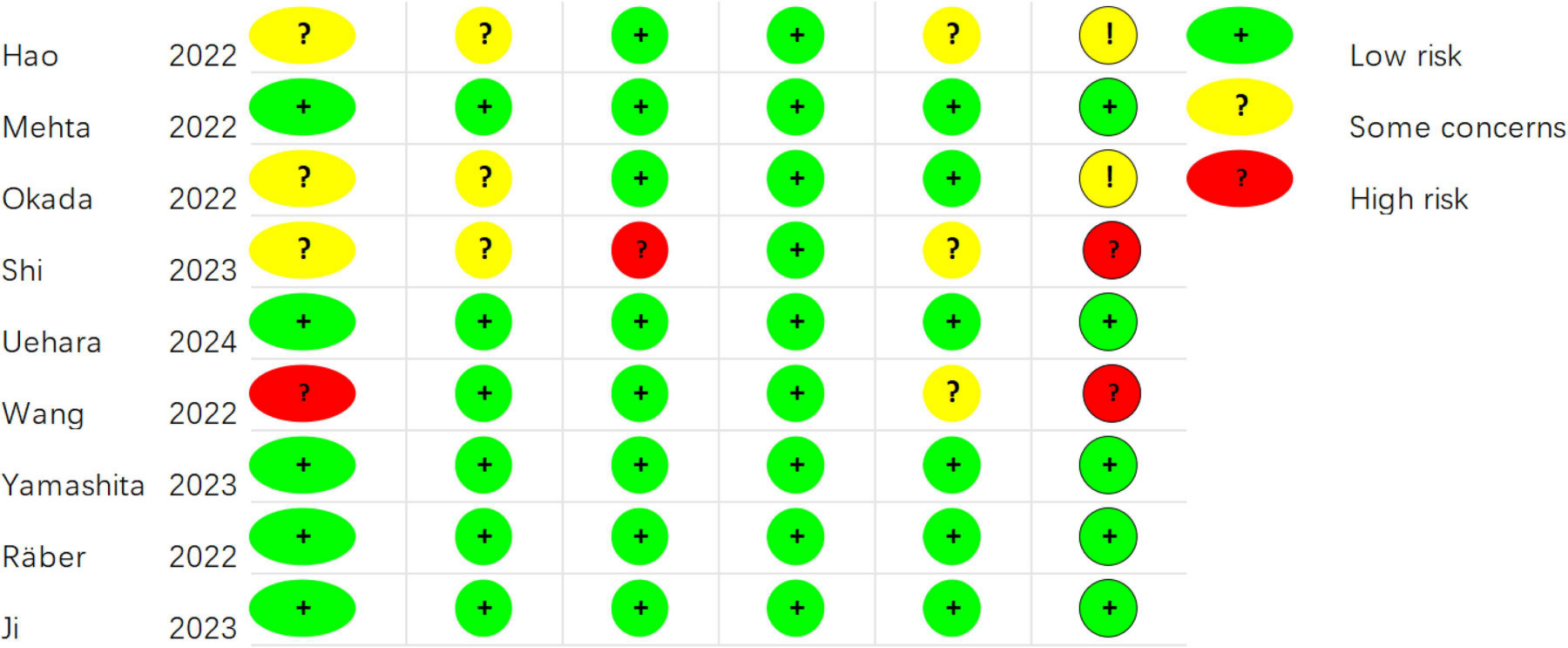
Risk of bias assessment for the included RCTs (RoB 2.0).

**Figure 3:**
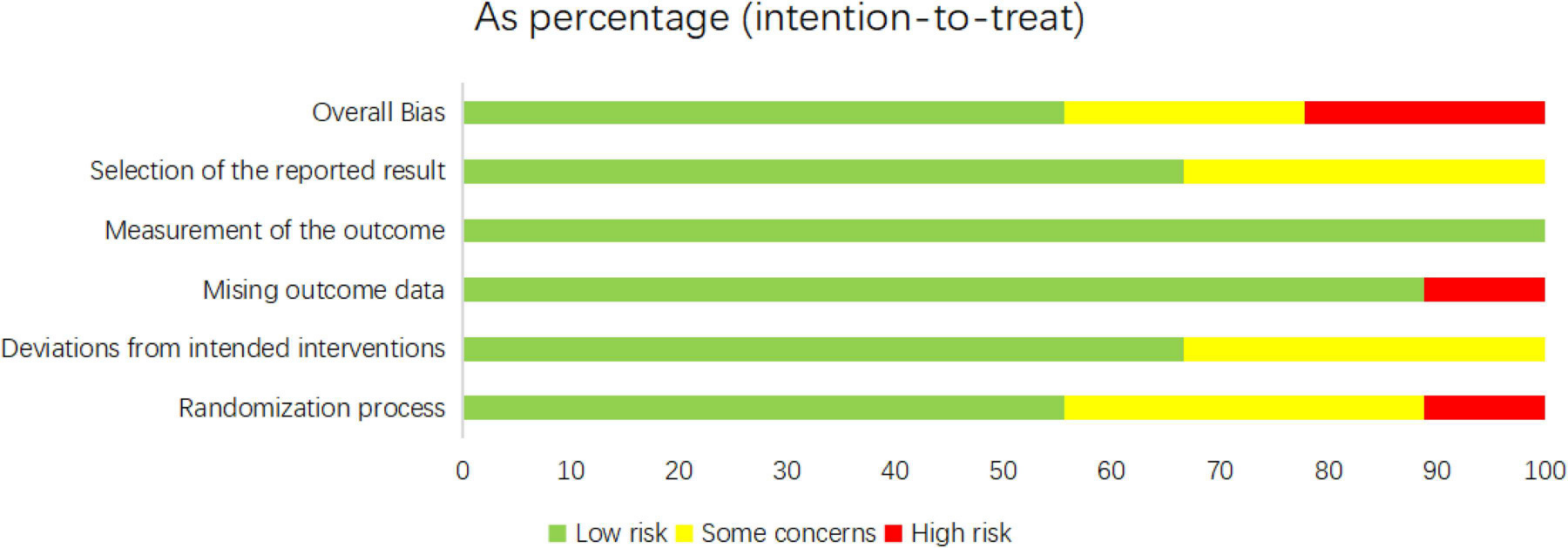
Risk of bias assessment summary for the included RCTs (RoB 2.0).

For the nine RCTs, the ROB was appraised by means of Cochrane ROB 2.0 (Figures 2, 3). For the randomization process, one study did not mention it, and three studies provided incomplete descriptions. Three studies were deemed at risk of deviating from intended interventions. One study had missing outcome data. Three studies showed the potential for selective reporting bias. Ultimately, five studies showed a low ROB, two exhibited a moderate ROB, and two demonstrated a high ROB. The NOS was leveraged to appraise the quality of cohort studies (Supplementary Table 2). Six articles were classified as high-quality studies, while two were categorized as medium to low-quality.

### 3.4 Study results

#### 3.4.1 MACE, non-fatal MI (NFMI), non-fatal stroke (NFS), and rehospitalization for unstable angina

**Figure 4:**
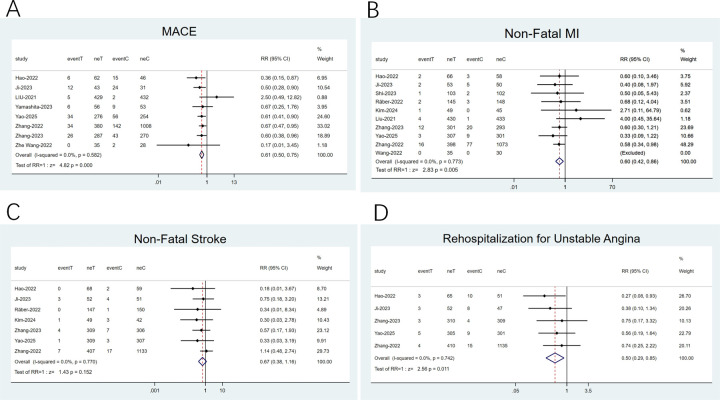
Forest plots: **(A)** MACE; **(B)** Non-fatal MI; **(C)** Non-fatal stroke; **(D)** rehospitalization for unstable angina.

Eight studies (20, 22, 23, 28–30, 34, 35) reported composite endpoints of MACE, ten (22–24, 26, 28–30, 32, 34, 35) documented NFMI, seven (22, 23, 26, 29, 32, 34, 35) addressed NFS, and five (22, 23, 29, 34, 35) examined readmissions for unstable angina. Minimal heterogeneity was noted (*I*^2^ < 50%), thereby employing a fixed-effects model. The meta-analysis implied that against the statin group, the PCSK9 inhibitor plus statin group reduced MACE incidence by 39% (RR: 0.61; 95% CI: 0.50–0.75; *p* < 0.001; Figure 4A), NFMI incidence by 40% (RR: 0.60; 95% CI: 0.42–0.86; *p* = 0.005; Figure 4B), and rates of rehospitalization for unstable angina by 50% (RR: 0.50; 95% CI: 0.29–0.85; *p* = 0.011; Figure 4D). Nonetheless, no apparent differences were noted in NFS incidence (RR: 0.67; 95% CI: 0.38–1.16; *p* = 0.15; Figure 4C).

#### 3.4.2 Unplanned revascularization, all-cause mortality (ACM), CVM, compliance rate of LDL-C ≤ 1.4 mmol/L

**Figure 5:**
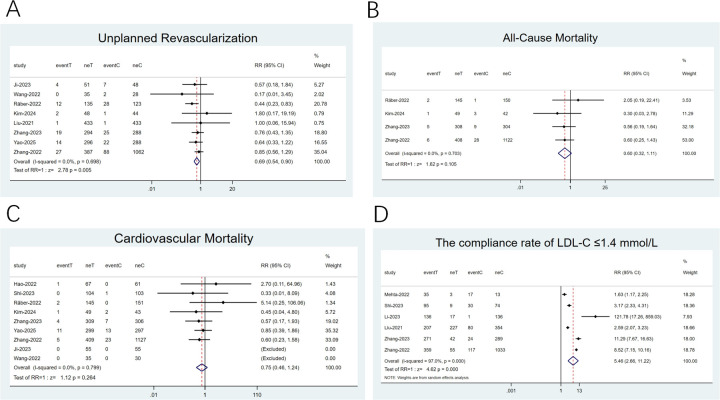
Forest plots: **(A)** unplanned revascularization; **(B)** All-cause mortality; **(C)** cardiovascular mortality; **(D)** the compliance rate of LDL-C ≤1.4 mmol/L.

Eight studies (22, 26, 28–30, 32, 34, 35) reported on unplanned revascularization, four (26, 32, 34, 35) focused on ACM, and nine (22–24, 26, 28, 29, 32, 34, 35) examined CVM. Low heterogeneity was noticed (*I*^2^ < 50%). Therefore, a fixed-effects model was employed. Unplanned revascularization risk was reduced by 31% with the combination therapy relative to statin monotherapy in the pooled analysis (RR: 0.69; 95% CI: 0.54–0.90; *p* = 0.005; Figure 5A). In contrast, the two groups exhibited no differences in ACM (RR: 0.60; 95% CI: 0.32–1.11; *p* = 0.105; Figure 5B) and CVM (RR: 0.75; 95% CI: 0.46–1.24; *p* = 0.26; Figure 5C). Six studies (21, 24, 30, 34–36) concentrated on the compliance rate of LDL-C ≤ 1.4 mmol/L, with remarkable heterogeneity (*I*^2^ = 97.0%, P < 0.001). Thus, a random-effects model was adopted. The results uncovered that the combination therapy resulted in a notable LDL-C compliance rate than statins alone (RR: 5.46; 95% CI: 2.66–11.22; *p* < 0.001; Figure 5D).

#### 3.4.3 LDL-C, lipoprotein(a) (Lp[a]), triglyceride (TG), and total cholesterol (TC)

**Figure 6:**
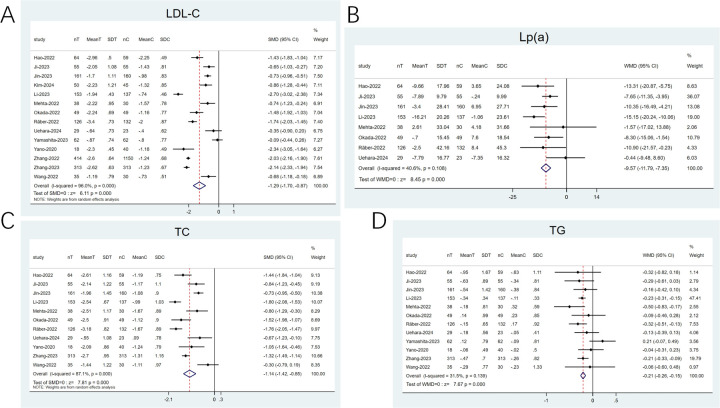
Forest plots: **(A)** LDL-C; **(B)** Lp(a); **(C)** TC; **(D)** TG.

14 articles (20–23, 25–28, 31–36) reported on LDL-C and 11 (21–23, 25–28, 31, 33, 35, 36) examined TC. High heterogeneity was noted (*I*^2^ ≥ 50%), and a random-effects model was applied. The SMD was utilized as the effect size. The pooled results revealed that the combination therapy exhibited a notable reduction in LDL-C (SMD: −1.29; 95% CI: −1.70 to −0.87; *p* < 0.001; Figure 6A) and TC (SMD: −1.14; 95% CI: −1.42 to −0.85; *p* < 0.001; Figure 6C) levels than statin monotherapy. 8 articles (21–23, 25–27, 31, 36) investigated LP(a) and 12 (20–23, 25–28, 31, 33, 35, 36) reported on TG. Low heterogeneity was observed (*I*^2^ < 50%), thereby utilizing a fixed-effects model. The findings revealed that in contrast to statins alone, the combination therapy exhibited a greater reduction in Lp(a) levels (WMD: −9.57; 95% CI: −11.79 to −7.35; *p* < 0.001; Figure 6B) and TG (WMD: −0.21; 95% CI: −0.26 to −0.15; *p* < 0.001; Figure 6D) levels.

#### 3.4.4 High-density lipoprotein cholesterol (HDL-C), non-HDL-C (NHDL-C), apoprotein (apo) A1 (apoA1), and apoB

**Figure 7:**
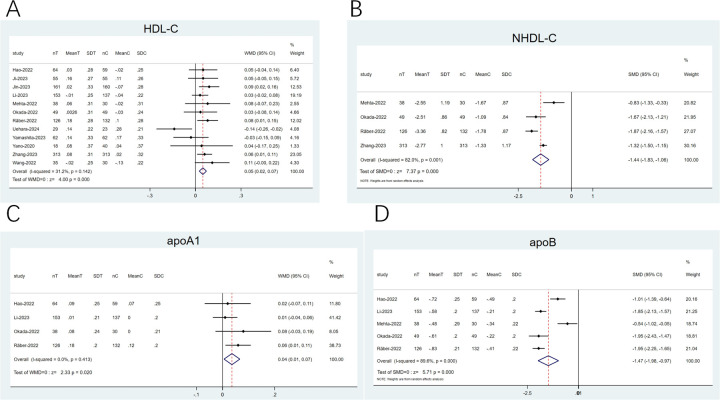
Forest plots: **(A)** HDL-C; **(B)** NHDL-C; **(C)** apoA1; **(D)** apoB.

12 articles (20–23, 25–28, 31, 33, 35, 36) reported on HDL-C, and 4 explored apoA1 (21, 25, 26, 35). The heterogeneity observed was low (*I*^2^ < 50%). Therefore, a fixed-effects model was opted for. The pooled analysis revealed that the combination therapy displayed a remarkable elevation in HDL-C (WMD: 0.05; 95% CI: 0.02–0.07; *p* < 0.001; Figure 7A) and apoA1 (WMD: 0.04; 95% CI: 0.01–0.07; *p* = 0.02; Figure 7C) levels than statins alone. Four articles (21, 25, 26) examined NHDL-C, and five articles (21, 23, 25, 26, 36) imported on apoB. The heterogeneity noted was noticeable (*I*^2^ ≥ 50%). Thus, a random-effects model was employed, with SMD as the effect size. The pooled results implied that against the statin group, the combination therapy group showed a greater decline in NHDL-C (SMD: −1.44; 95% CI: −1.83 to −1.06; *p* < 0.001; Figure 7B) and apoB (SMD: −1.47; 95% CI: −1.98 to −0.97; *p* < 0.001; Figure 7D) levels.

### 3.5 Sensitivity analysis

The leave-one-out method was leveraged for sensitivity analysis by systematically removing each study to identify sources of heterogeneity in high-heterogeneity outcomes and assess the robustness of the findings.

Following the exclusion of the study by Mehta et al. (21) resulted in a reduction in the heterogeneity for NHDL-C (*I*^2^ = 0.0%, P = 0.542). Moreover, the effect size of the results before and after exclusion showed no notable change (WMD: −1.49; 95% CI: −1.61 to −1.36; *P* < 0.001; Figure 8). This might be explained by the fact that their study utilized alirocumab, whereas other studies employed evolocumab. The remaining studies did not have a noticeable impact on the overall results, thereby confirming the robustness and reliability of our findings (Supplementary Figures 1–15).

**Figure 8:**
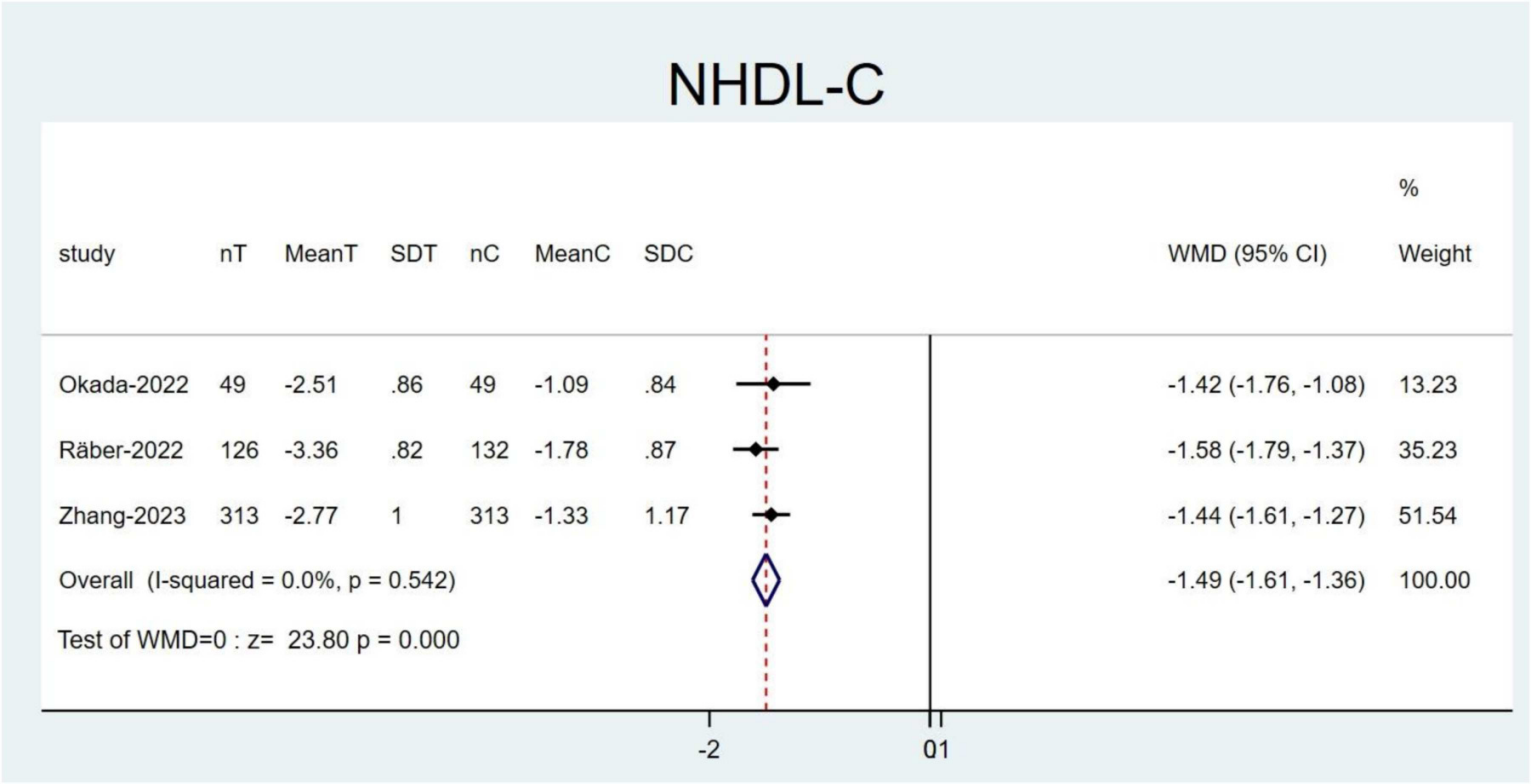
Forest plots: NHDL-C result after excluding the study by Mehta et al.

### 3.6 Subgroup analysis and meta-regression analysis

Considering the remarkable impact of study type, intervention measures, and disease types on high heterogeneity results, subgroup analyses were conducted based on these factors (Supplementary Figures 16–27). Nonetheless, no source of heterogeneity was identified. This could possibly be explained by the small number of studies included or the insufficient sample sizes.

Therefore, a meta-regression analysis was executed to recognize the source of heterogeneity in highly heterogeneous studies (Supplementary Figures 28–34). The results implied that the heterogeneity of the results in this study primarily arose from differences among the studies in terms of study type, intervention measures, types of underlying diseases, baseline levels, and sample sizes. For instance, the heterogeneity in the compliance rate of LDL-C levels (Tau2 = 0.6451) primarily stemmed from study type (Tau2 = 0.0085, *P* = 0.044) and intervention measures (Tau2 = 0.0085, *P* = 0.030). Based on the variation in Tau2, these factors accounted for 99% of the sources of heterogeneity.

### 3.7 Publication bias

Publication bias was appraised for outcomes mentioned in at least 10 studies by means of Egger's test, Begg's test, visual inspection on funnel plots, and the imputation method (Supplementary Figures 35–39). The results indicated no publication bias, further reinforcing the robustness and reliability of our findings.

### 3.8 GRADE quality assessment

Using the GRADE framework, we executed a comprehensive assessment of the evidence quality for every outcome (19) (Table 2). Overall, the quality of evidence across outcomes ranged from moderate to low. The evidence for the combination therapy in reducing MACE and improving lipid profiles is well-established.

**Table 2:** GRADE quality of evidence for outcomes.

Outcome	Risk of bias	Inconsistency	Indirectness	Imprecision	Publication bias	Quality of evidence
MACE	High	None	None	None	None	Moderate
Cardiovascular Mortality	High	None	None	Yes	None	Low
Non-fatal MI	High	None	None	None	None	Moderate
Non-fatal Stroke	High	None	None	Yes	None	Low
Rehospitalization for Unstable Angina	High	None	None	None	None	Moderate
Unplanned Revascularization	High	None	None	None	None	Moderate
All-cause Mortality	Low	None	None	Yes	None	Moderate
LDL-C level	High	None	None	Yes	None	Low
LDL-C compliance rate	Low	None	None	None	None	Moderate
Lp(a)	Low	None	None	None	None	Moderate
TC	Low	None	None	Yes	None	Very Low
TG	Low	None	None	None	None	Low
HDL-C	Low	None	None	Yes	None	Low
NHDL-C	Low	None	None	None	None	Moderate
apoB	Low	None	None	None	None	Very Low
apoA1	Low	None	None	None	None	Low
hs-CRP	High	None	None	Yes	None	Low

MACE, major adverse cardiovascular events; RCT, randomized controlled trial; MI, myocardial infarction.

## 4 Discussion

The functional deficiency of PCSK9 may confer a protective effect against CVDs (37). PCSK9 inhibitors increase the number of LDLR on hepatocyte surfaces by blocking the binding of PCSK9 to LDLR, thereby preventing PCSK9-induced LDLR degradation. This enhances the capacity of the liver to clear LDL-C (38). PCSK9 inhibitors have recently arisen as an innovative therapeutic approach for CVDs due to their ability to simultaneously lower lipid levels (39, 40). Multiple large-scale clinical outcome trials have confirmed that these inhibitors can diminish MACE risks by approximately 15% (41, 42). Additionally, they can improve coronary microcirculation and cardiac function in post-PCI patients (43–45). Nonetheless, no systematic review or meta-analysis specifically targets the cardiovascular outcomes for PCSK9 inhibitors in patients after PCI.

### 4.1 Main findings

The study represents the first systematic review and meta-analysis to appraise the overall impact of PCSK9 inhibitors in combination with statins on cardiovascular outcomes, lipid management, and plaque stability in patients following PCI. Through the analysis of 17 studies involving 5,607 patients, it is found that compared to statin therapy alone, combining PCSK9 inhibitors with statins noticeably reduces MACE risks in patients following PCI. Moreover, the incidences of NFMI, rehospitalization for unstable angina, and unplanned revascularization are also decreased. In contrast, regarding the incidence of NFS, ACM, and CVM, the combination therapy shows no notable difference when compared to statin therapy. Meanwhile, several prior studies targeting ACS and ASCVD populations have verified the role of PCSK9 inhibitors in lowering cardiovascular risk (46–48), strongly supporting the conclusions of this study.

In lipid control, LDL-C is a key factor in atherosclerosis. Its abnormal accumulation in the vascular endothelium can induce oxidative stress and inflammatory responses, leading to plaque progression. In this study, the combination therapy leads to a 1.29 standard deviation reduction in LDL-C levels, with the compliance rate of LDL-C (≤1.4 mmol/L) increasing by nearly six times, remarkably outperforming the effects of statin monotherapy in prior studies. It has been found that for each 1 mmol/L reduction in LDL-C, MACE risks can be decreased by 22%–24% (49). This is in strong agreement with the observed decline in LDL-C levels and MACE risks in our study, further confirming the superiority of the combination therapy. Furthermore, NHDL-C encompasses all atherogenic lipoproteins, covering LDL, very LDL, and intermediate-density lipoprotein. apoB can directly reflect the total number of these lipoproteins. The 2019 ESC guidelines indicate that apoB is superior to LDL-C in predicting cardiovascular risks (8), demonstrating that it might serve as a more sensitive indicator for examining the efficacy of lipid-lowering therapies. In this study, apoB levels are noticeably reduced (SMD = −1.47), indicating a decrease in the total number of atherogenic particles. Concurrently, the marked decline in NHDL-C (WMD = −1.49 mmol/L) suggests a reduction in total cholesterol burden. Together, the two findings corroborate an improvement in lipid metabolism. Research indicates that lowering apoB to a more stringent target (like <65 mg/dl) can further inhibit the progression of arterial plaques and reduce cardiovascular events (50). This provides noticeable evidence for the implementation of intensified lipid-lowering therapy. HDL-C inhibits atherosclerosis by removing cholesterol from the vascular wall through reverse cholesterol transport, while apoA1, as the primary structural protein of HDL, shares a similar mechanism in reducing the risk of atherosclerosis. It has been proven that the levels of HDL-C and apoA1 are notably negatively tied to the progression of atherosclerotic plaques (51, 52). Our findings reveal that elevated HDL-C and apoA1 concentrations are noted in patients receiving combination therapy relative to those on statin treatment alone. This suggests that PCSK9 inhibitors may slow down plaque progression in patients after PCI by elevating these levels. Moreover, increased Lp(a) levels are recognized as an independent risk factor for CVDs. Despite achieving target LDL-C levels, high Lp(a) levels can still increase cardiovascular risk (53). A previous study has implied that PCSK9 inhibitors can decrease LP(a) levels by 25% to 30% (54). Our research corroborates these findings. An epidemiological study reveals a connection between elevated TC and TG levels and a higher risk of cardiovascular events. Conversely, the decline in TC and TG levels can decrease the cardiovascular risk for patients (55). This study suggests that the combination therapy group has lower TC and TG levels, indicating that PCSK9 inhibitors can diminish cardiovascular risk by reducing these lipid levels. In summary, compared to monotherapy with statins, the combination therapy demonstrates a noticeable advantage in improving lipid profiles in patients following PCI. Furthermore, our findings in post-PCI patients are corroborated by studies in other high-risk populations, such as those with Familial Hypercholesterolemia (FH). For instance, Scicali et al. demonstrated in 56 FH subjects that adding a PCSK9 inhibitor led to a comparable LDL-C reduction of 49.61% while also improving markers of inflammation and arterial stiffness (PWV) (56). Offering a deeper mechanistic perspective. Toscano et al. found in 26 FH subjects that circulating PCSK9 levels directly correlate with PWV. Their research highlighted that adding a PCSK9 inhibitor overcomes the paradoxical statin-induced rise in PCSK9, leading to reductions in both PCSK9 levels and PWV (57). Collectively, this robust evidence from FH patients reinforces our conclusion that combination therapy is a highly effective strategy for managing cardiovascular risk, likely through both LDL-C-dependent and independent vascular mechanisms.

Moreover, despite the clear clinical efficacy of PCSK9 inhibitors, cost-effectiveness studies show that these medications are generally not cost-effective in high-income countries (58). In the context of less developed countries (like China), even with a 70% decline in the annual cost of PCSK9 inhibitors through national health insurance negotiations, the incremental cost-effectiveness ratio remains well above China's willingness-to-pay threshold, which is three-fold the per capita GDP (59). In order to satisfy the traditional willingness-to-pay threshold, the price of PCSK9 inhibitors would need to be lowered by 20% to 86% (58). This prevents many high-risk patients from accessing optimal treatment due to financial constraints.

### 4.2 Limitations

This study has several limitations. It includes both RCTs and cohort studies. Among the nine RCTs included, two have a high ROB. Additionally, in the cohort studies, two have an NOS score <7. Thus, the robustness of the conclusions might be influenced.

In terms of interventions, different studies exhibit variations in the type of PCSK9 inhibitors (like evolocumab and alirocumab) and their doses (such as 140 mg Q2W and 420 mg QM), as well as the type of statins (like atorvastatin and rosuvastatin) and their doses (such as high/moderate doses), which may lead to potential heterogeneity. Although subgroup analyses are performed to examine the dose-response relationship, due to the limited number of included articles, the influence of different regimens on the outcomes is not clarified. Finally, our meta-analysis did not include data on the novel small interfering RNA (siRNA) therapeutic, inclisiran. At the time of our literature search, no suitable randomized controlled trials with long-term follow-up reporting its effects on MACE or lipid levels outcomes were available for inclusion.

For outcome measures, some studies define MACE as a composite of CVM, NFMI, and rehospitalization for unstable angina, while other studies include unplanned revascularization or NFS. This discrepancy may lead to heterogeneity in the results.

During the systematic review, as this study only includes English-language articles, important studies in other languages may have been missed. Additionally, only two studies have a follow-up duration of ≥2 years. Atherosclerosis is a lifelong progressive disease. The absence of long-term follow-up data may result in an underestimation of the benefits of the combination therapy on ACM and CVM.

### 4.3 Insights for future practice and research

Our study confirms the efficacy of PCSK9 inhibitors combined with statins for patients undergoing PCI. However, noticeable differences are observed in the dosages of the drugs used across various studies. Future studies should explore the dose-response relationship to assess whether high doses (like evolocumab 420 mg QM) can further reduce cardiovascular risk or lipid levels. Future multicenter, large-sample RCTs with extended follow-up periods of at least five years are needed to reassess the combination therapy's impact on all-cause mortality (ACM) and cardiovascular mortality (CVM), as well as to validate the long-term efficacy of standard maintenance doses (such as evolocumab 140 mg Q2W). Moreover, with the emergence of novel therapies like inclisiran, which has already shown efficacy in improving lipid and vascular profiles (60), future large-scale, long-term RCTs are also urgently warranted. Such trials should be designed to investigate the efficacy of an inclisiran-statin combination therapy on hard clinical endpoints like MACE, ACM, and cardiovascular mortality.

Since PCSK9 inhibitors are not dependent on the hepatic CYP450 enzyme for metabolism, they may offer therapeutic advantages in patients with liver dysfunction or elevated baseline of ALT. These agents could be considered a preferred treatment option for such populations in the future. However, data regarding hepatic safety in patients with chronic liver disease remain limited. Further evaluation through multicenter RCTs is necessary to assess their impact on hepatic metabolism and drug interactions, thereby clarifying the applicability of PCSK9 inhibitors within this patient group.

This study finds that LDL-C, apoB, apoA1, HDL-C, and NHDL-C are notably linked to cardiovascular risk. To enhance the comprehensiveness of cardiovascular risk assessment, future efforts might focus on integrating these indicators to establish a composite evaluation system. For example, the apoB/apoA1 ratio (BAR) reflects the imbalance between atherogenic lipoprotein (ApoB) and anti-atherogenic lipoprotein (ApoA1). An increase in this ratio can exacerbate plaque formation by promoting lipid deposition and inflammatory responses. Research indicates that for each 1 standard deviation increase in the BAR, the risk of CAD rises by 29.1% (61). Similarly, the NHDL-C/HDL-C ratio (NHHR) reflects the balance between atherogenic (NHDL-C) and protective (HDL-C) lipoproteins. An increase of 1 unit in NHHR leads to a 23% rise in CVD risk (62). This ratio can better assess the impact of dyslipidemia on cardiovascular health. BAR and NHHR are independent risk factors for coronary artery disease, with predictive performance that does not rely on traditional lipid indicators (LDL-C). Therefore, future research could integrate BAR, NHHR, and biomarkers like LDL-C and Lp(a) to multidimensionally assess cardiovascular risk, thereby enhancing predictive accuracy.

## 5 Conclusion

The combination of PCSK9 inhibitors and statins can noticeably decrease MACE risks in patients following PCI. Additionally, this combination therapy markedly improves lipid profiles. Given the heterogeneity of intervention protocols, ROBs, and short-term follow-up data, future studies should standardize doses, extend follow-up, and incorporate more studies to recover the influence of combination therapy on long-term mortality and other risks in clinical practice.

## Data Availability

The original contributions presented in the study are included in the article/Supplementary Material, further inquiries can be directed to the corresponding author.
