# Supplementary material for: Effectiveness of combining PCSK9 inhibitors with statins on major adverse cardiovascular events and lipid levels in patients after percutaneous coronary intervention: a systematic review and meta-analysis
Source: Front Cardiovasc Med. 2025 Oct 29;12:1612095. doi: 10.3389/fcvm.2025.1612095 (PMC12605067; doi:10.3389/fcvm.2025.1612095)
Supplement: Supplementary file 1 [file Datasheet1.pdf]

Supplementary Materials

Supplementary Tables

Supplementary Table 1 Search strategies

Pubmed

Serial	Search strategy	Result
#1	Percutaneous Coronary Intervention [MeSH Terms]	68,786
#2	"Percutaneous Coronary Intervention"[Title/Abstract] OR "Coronary Intervention, Percutaneous"[Title/Abstract] OR "Coronary Interventions, Percutaneous"[Title/Abstract] OR "Intervention, Percutaneous Coronary"[Title/Abstract] OR "Interventions, Percutaneous Coronary"[Title/Abstract] OR "Percutaneous Coronary Interventions"[Title/Abstract] OR "Percutaneous Coronary Revascularization"[Title/Abstract] OR "Coronary Revascularization, Percutaneous"[Title/Abstract] OR "Coronary Revascularizations, Percutaneous"[Title/Abstract] OR "Percutaneous Coronary Revascularizations"[Title/Abstract] OR "Revascularization, Percutaneous Coronary"[Title/Abstract] OR "Revascularizations, Percutaneous Coronary"[Title/Abstract]	51,130
#3	Hydroxymethylglutaryl-CoA Reductase Inhibitors[MeSH Terms]	36,424
#4	"Hydroxymethylglutaryl CoA Reductase Inhibitors"[Title/Abstract] OR "Inhibitors, Hydroxymethylglutaryl-CoA Reductase"[Title/Abstract] OR "Reductase Inhibitors, Hydroxymethylglutaryl-CoA"[Title/Abstract] OR "HMG-CoA Reductase Inhibitor"[Title/Abstract] OR "HMG CoA Reductase Inhibitor"[Title/Abstract] OR "Statin"[Title/Abstract] OR "Statins"[Title/Abstract] OR "Inhibitors, HMG-CoA Reductase"[Title/Abstract] OR "Inhibitors, HMG CoA Reductase"[Title/Abstract] OR "Reductase Inhibitors, HMG-CoA"[Title/Abstract] OR "HMG-CoA Reductase Inhibitors"[Title/Abstract] OR "HMG CoA Reductase Inhibitors"[Title/Abstract] OR "Inhibitors, Hydroxymethylglutaryl-Coenzyme A"[Title/Abstract] OR "Hydroxymethylglutaryl-Coenzyme A Inhibitors"[Title/Abstract] OR "Inhibitors, Hydroxymethylglutaryl Coenzyme A"[Title/Abstract] OR "Inhibitors, Hydroxymethylglutaryl-CoA"[Title/Abstract] OR "Hydroxymethylglutaryl-CoA Inhibitors"[Title/Abstract] OR "Inhibitors, Hydroxymethylglutaryl CoA"[Title/Abstract] OR "Hydroxymethylglutaryl-CoA Reductase Inhibitor"[Title/Abstract] OR "Hydroxymethylglutaryl CoA Reductase Inhibitor"[Title/Abstract] OR "Reductase Inhibitor, Hydroxymethylglutaryl-CoA"[Title/Abstract] OR "Statins, HMG-CoA"[Title/Abstract] OR "HMG-CoA Statins"[Title/Abstract] OR "Statins, HMG CoA"[Title/Abstract] OR "Cerivastatin "[Title/Abstract] OR "Atorvastatin"[Title/Abstract] OR "Lipitor"[Title/Abstract] OR "Rosuvastatin"[Title/Abstract] OR "Crestor"[Title/Abstract] OR "Simvastatin"[Title/Abstract] OR "Zocor"[Title/Abstract] OR "Pravastatin"[Title/Abstract] OR "Pravachol"[Title/Abstract] OR "Lovastatin"[Title/Abstract] OR "Mevacor"[Title/Abstract] OR	69,678

	"Fluvastatin"[Title/Abstract] OR "Lescol"[Title/Abstract] OR "Pitavastatin"[Title/Abstract] OR "Livalo"[Title/Abstract]	
#5	PCSK9 Inhibitors[MeSH Terms]	1,402
#6	"PCSK9 Inhibitors, Cardiovascular"[Title/Abstract] OR "Cardiovascular PCSK9 Inhibitors"[Title/Abstract] OR "Inhibitors, Cardiovascular PCSK9"[Title/Abstract] OR "PCSK9 Inhibitors Cardiovascular"[Title/Abstract] OR "Cardiovascular, PCSK9 Inhibitors"[Title/Abstract] OR "Evolocumab"[Title/Abstract] OR "Repatha"[Title/Abstract] OR "Alirocumab"[Title/Abstract] OR "Praluent"[Title/Abstract] OR "Inclisiran"[Title/Abstract] OR "Leqvio"[Title/Abstract]	1,872
#7	(#1 OR #2) AND (#3 OR #4) AND (#5 OR #6)	46

Embase

Serial	Search strategy	Result
#1	'percutaneous coronary intervention'/exp	140,215
#2	'percutaneous coronary intervention':ab,ti,kw	80,110
#3	'hydroxymethylglutaryl coenzyme a reductase inhibitor'/exp	208,155
#4	'hmg coa reductase inhibitor':ab,ti,kw OR 'hmg coa reductase inhibitors':ab,ti,kw OR 'hmg coenzyme a reductase inhibitor':ab,ti,kw OR 'hmg-coa reductase inhibitors':ab,ti,kw OR 'hydroxymethylglutaryl coa reductase inhibitors':ab,ti,kw OR 'hydroxymethylglutaryl-coa reductase inhibitors':ab,ti,kw OR 'statin drug':ab,ti,kw OR 'statins':ab,ti,kw OR 'hydroxymethylglutaryl coenzyme a reductase inhibitor':ab,ti,kw OR 'cerivastatin':ab,ti,kw OR 'atorvastatin':ab,ti,kw OR 'lipitor':ab,ti,kw OR 'rosuvastatin':ab,ti,kw OR 'crestor':ab,ti,kw OR 'simvastatin':ab,ti,kw OR 'zocor':ab,ti,kw OR 'pravastatin':ab,ti,kw OR 'pravachol':ab,ti,kw OR 'lovastatin':ab,ti,kw OR 'mevacor':ab,ti,kw OR 'fluvastatin':ab,ti,kw OR 'lescol':ab,ti,kw OR 'pitavastatin':ab,ti,kw OR 'livalo':ab,ti,kw	93,810
#5	'pcsk9 inhibitor'/exp	6,898
#6	'pcsk9 inhibitors':ab,ti,kw OR 'pcsk9 inhibitor':ab,ti,kw OR 'evolocumab':ab,ti,kw OR 'repatha':ab,ti,kw OR 'alirocumab':ab,ti,kw OR 'praluent':ab,ti,kw OR 'inclisiran':ab,ti,kw OR 'leqvio':ab,ti,kw	5,555
#7	('percutaneous coronary intervention'/exp OR 'percutaneous coronary intervention':ab,ti,kw) AND ('hydroxymethylglutaryl coenzyme a reductase inhibitor'/exp OR ('hmg coa reductase inhibitor':ab,ti,kw OR 'hmg coa reductase inhibitors':ab,ti,kw OR 'hmg coenzyme a reductase inhibitor':ab,ti,kw OR 'hmg-coa reductase inhibitors':ab,ti,kw OR 'hydroxymethylglutaryl coa	301

	reductase inhibitors':ab,ti,kw OR 'hydroxymethylglutaryl-coa reductase inhibitors':ab,ti,kw OR 'statin drug':ab,ti,kw OR 'statins':ab,ti,kw OR 'hydroxymethylglutaryl coenzyme a reductase inhibitor':ab,ti,kw OR 'cerivastatin':ab,ti,kw OR 'atorvastatin':ab,ti,kw OR 'lipitor':ab,ti,kw OR 'rosuvastatin':ab,ti,kw OR 'crestor':ab,ti,kw OR 'simvastatin':ab,ti,kw OR 'zocor':ab,ti,kw OR 'pravastatin':ab,ti,kw OR 'pravachol':ab,ti,kw OR 'lovastatin':ab,ti,kw OR 'mevacor':ab,ti,kw OR 'fluvastatin':ab,ti,kw OR 'lescol':ab,ti,kw OR 'pitavastatin':ab,ti,kw OR 'livalo':ab,ti,kw)) AND ('pcsk9 inhibitor'/exp OR ('pcsk9 inhibitors':ab,ti,kw OR 'pcsk9 inhibitor':ab,ti,kw OR 'evolocumab':ab,ti,kw OR 'repatha':ab,ti,kw OR 'alirocumab':ab,ti,kw OR 'praluent':ab,ti,kw OR 'inclisiran':ab,ti,kw OR 'leqvio':ab,ti,kw))	

Cochrane Library

Serial	Search strategy	Result
#1	MeSH descriptor: [Percutaneous Coronary Intervention] explode all trees	87,180
#2	(‘Coronary Revascularization, Percutaneous' OR ' Revascularization, Percutaneous Coronary' OR ' Percutaneous Coronary Revascularizations' OR ' Interventions, Percutaneous Coronary' OR ' Percutaneous Coronary Revascularization' OR ' Coronary Intervention, Percutaneous' OR ' Coronary Interventions, Percutaneous' OR ' Revascularizations, Percutaneous Coronary' OR ' Intervention, Percutaneous Coronary' OR ' Coronary Revascularizations, Percutaneous' OR ' Percutaneous Coronary Interventions’):ti,ab,kw	14,342
#3	MeSH descriptor: [Hydroxymethylglutaryl-CoA Reductase Inhibitors] explode all trees	4,971
#4	(‘Reductase Inhibitor, Hydroxymethylglutaryl-CoA' OR ' Inhibitors, HMG-CoA Reductase' OR ' Inhibitors, HMG CoA Reductase' OR ' Hydroxymethylglutaryl-Coenzyme A Inhibitors' OR ' Hydroxymethylglutaryl CoA Reductase Inhibitor' OR ' Reductase Inhibitors, HMG-CoA' OR ' Inhibitors, Hydroxymethylglutaryl Coenzyme A' OR ' HMG-CoA Reductase Inhibitor' OR ' Hydroxymethylglutaryl-CoA Reductase Inhibitor' OR ' HMG CoA Reductase Inhibitor' OR ' Inhibitors, Hydroxymethylglutaryl-CoA Reductase' OR ' HMG-CoA Reductase Inhibitors' OR ' Reductase Inhibitors, Hydroxymethylglutaryl-CoA' OR ' HMG CoA Reductase Inhibitors' OR ' Hydroxymethylglutaryl CoA Reductase Inhibitors' OR ' Inhibitors, Hydroxymethylglutaryl-Coenzyme A' OR ' Statins, HMG CoA' OR ' Statins, HMG-CoA' OR ' HMG-CoA Statins' OR ' Hydroxymethylglutaryl-CoA Inhibitors' OR ' Inhibitors, Hydroxymethylglutaryl CoA' OR ' Inhibitors, Hydroxymethylglutaryl-CoA' OR ' Statins' OR ' Statin' OR ‘Cerivastatin ' OR 'Atorvastatin' OR 'Lipitor' OR 'Rosuvastatin' OR 'Crestor' OR 'Simvastatin' OR 'Zocor' OR 'Pravastatin' OR 'Pravachol' OR 'Lovastatin' OR 'Mevacor' OR 'Fluvastatin' OR 'Lescol' OR 'Pitavastatin' OR 'Livalo’):ti,ab,kw	19,977
#5	MeSH descriptor: [PCSK9 Inhibitors] explode all trees	183

#6	(‘Cardiovascular, PCSK9 Inhibitors' OR ' Cardiovascular PCSK9 Inhibitors' OR ' Inhibitors, Cardiovascular PCSK9' OR ' PCSK9 Inhibitors Cardiovascular' OR ' PCSK9 Inhibitors, Cardiovascular' OR 'Evolocumab' OR 'Repatha' OR 'Alirocumab' OR 'Praluent' OR 'Inclisiran' OR 'Leqvio'):ti,ab,kw	1,151
#7	(#1 OR #2) AND (#3 OR #4) AND (#5 OR #6)	44

Web of Science

Serial	Search strategy	Result
#1	Percutaneous Coronary Intervention (All Fields) OR "Percutaneous Coronary Intervention" OR "Coronary Intervention, Percutaneous" OR "Coronary Interventions, Percutaneous" OR "Intervention, Percutaneous Coronary" OR "Interventions, Percutaneous Coronary" OR "Percutaneous Coronary Interventions" OR "Percutaneous Coronary Revascularization" OR "Coronary Revascularization, Percutaneous" OR "Coronary Revascularizations, Percutaneous" OR "Percutaneous Coronary Revascularizations" OR "Revascularization, Percutaneous Coronary" OR "Revascularizations, Percutaneous Coronary" (Title) OR "Percutaneous Coronary Intervention" OR "Coronary Intervention, Percutaneous" OR "Coronary Interventions, Percutaneous" OR "Intervention, Percutaneous Coronary" OR "Interventions, Percutaneous Coronary" OR "Percutaneous Coronary Interventions" OR "Percutaneous Coronary Revascularization" OR "Coronary Revascularization, Percutaneous" OR "Coronary Revascularizations, Percutaneous" OR "Percutaneous Coronary Revascularizations" OR "Revascularization, Percutaneous Coronary" OR "Revascularizations, Percutaneous Coronary" (Abstract)	70,441
#2	Hydroxymethylglutaryl-CoA Reductase Inhibitors (All Fields) OR "Hydroxymethylglutaryl CoA Reductase Inhibitors" OR "Inhibitors, Hydroxymethylglutaryl-CoA Reductase" OR "Reductase Inhibitors, Hydroxymethylglutaryl-CoA" OR "HMG-CoA Reductase Inhibitor" OR "HMG CoA Reductase Inhibitor" OR "Statin" OR "Statins" OR "Inhibitors, HMG-CoA Reductase" OR "Inhibitors, HMG CoA Reductase" OR "Reductase Inhibitors, HMG-CoA" OR "HMG-CoA Reductase Inhibitors" OR "HMG CoA Reductase Inhibitors" OR "Inhibitors, Hydroxymethylglutaryl-Coenzyme A" OR "Hydroxymethylglutaryl-Coenzyme A Inhibitors" OR "Inhibitors, Hydroxymethylglutaryl Coenzyme A" OR "Inhibitors, Hydroxymethylglutaryl-CoA" OR "Hydroxymethylglutaryl-CoA Inhibitors" OR "Inhibitors, Hydroxymethylglutaryl CoA" OR "Hydroxymethylglutaryl-CoA Reductase Inhibitor" OR "Hydroxymethylglutaryl CoA Reductase Inhibitor" OR "Reductase Inhibitor, Hydroxymethylglutaryl-CoA" OR "Statins, HMG-CoA" OR "HMG-CoA Statins" OR "Statins, HMG CoA" OR "Cerivastatin " OR "Atorvastatin" OR "Lipitor" OR "Rosuvastatin" OR "Crestor" OR "Simvastatin" OR "Zocor" OR "Pravastatin" OR "Pravachol" OR "Lovastatin"	78,286

	OR "Mevacor" OR "Fluvastatin" OR "Lescol" OR "Pitavastatin" OR "Livalo" (Title) OR "Hydroxymethylglutaryl CoA Reductase Inhibitors" OR "Inhibitors, Hydroxymethylglutaryl-CoA Reductase" OR "Reductase Inhibitors, Hydroxymethylglutaryl-CoA" OR "HMG-CoA Reductase Inhibitor" OR "HMG CoA Reductase Inhibitor" OR "Statin" OR "Statins" OR "Inhibitors, HMG-CoA Reductase" OR "Inhibitors, HMG CoA Reductase" OR "Reductase Inhibitors, HMG-CoA" OR "HMG-CoA Reductase Inhibitors" OR "HMG CoA Reductase Inhibitors" OR "Inhibitors, Hydroxymethylglutaryl-Coenzyme A" OR "Hydroxymethylglutaryl-Coenzyme A Inhibitors" OR "Inhibitors, Hydroxymethylglutaryl Coenzyme A" OR "Inhibitors, Hydroxymethylglutaryl-CoA" OR "Hydroxymethylglutaryl-CoA Inhibitors" OR "Inhibitors, Hydroxymethylglutaryl CoA" OR "Hydroxymethylglutaryl-CoA Reductase Inhibitor" OR "Hydroxymethylglutaryl CoA Reductase Inhibitor" OR "Reductase Inhibitor, Hydroxymethylglutaryl-CoA" OR "Statins, HMG-CoA" OR "HMG-CoA Statins" OR "Statins, HMG CoA" OR "Cerivastatin " OR "Atorvastatin" OR "Lipitor" OR "Rosuvastatin" OR "Crestor" OR "Simvastatin" OR "Zocor" OR "Pravastatin" OR "Pravachol" OR "Lovastatin" OR "Mevacor" OR "Fluvastatin" OR "Lescol" OR "Pitavastatin" OR "Livalo" (Abstract)	
#3	PCSK9 Inhibitors (All Fields) OR "PCSK9 Inhibitors, Cardiovascular" OR "Cardiovascular PCSK9 Inhibitors" OR "Inhibitors, Cardiovascular PCSK9" OR "PCSK9 Inhibitors Cardiovascular" OR "Cardiovascular, PCSK9 Inhibitors" OR "Evolocumab" OR "Repatha" OR "Alirocumab" OR "Praluent" OR "Inclisiran" OR "Leqvio" (Title) OR "PCSK9 Inhibitors, Cardiovascular" OR "Cardiovascular PCSK9 Inhibitors" OR "Inhibitors, Cardiovascular PCSK9" OR "PCSK9 Inhibitors Cardiovascular" OR "Cardiovascular, PCSK9 Inhibitors" OR "Evolocumab" OR "Repatha" OR "Alirocumab" OR "Praluent" OR "Inclisiran" OR "Leqvio" (Abstract	4,464
#4	#1 AND (#2 AND #3)	46

Supplementary Table 2 Risk of bias in Cohort Studies

Study	Selection (Score each entry 0 to 1)				Comparability (Maximum 2 points)	Exposure (Score each entry 0 to 1)			Totals
	Representativeness of the exposed cohort	Selection of the non exposed cohort	Ascertainment of exposure	Demonstration that outcome of interest was not present at start of study	Comparability of cohorts on the basis of the design or analysis	Assessment of outcome	Was follow up long enough for outcomes to occur	Adequacy of follow up of cohorts	
Kim-2024	1	1	1	1	1	0	1	1	7
Li-2023	1	1	1	0	1	1	0	1	6
Liu-2021	1	1	1	0	1	0	1	1	6
Jin-2023	1	1	1	1	2	1	1	0	8
Yano-2020	1	1	1	1	1	1	1	1	8
Zhang-	1	1	1	1	2	1	1	1	9

2023									
Yao-2025	1	1	1	1	2	0	1	1	8
Zhang-2022	1	1	1	1	2	0	1	1	8

Supplementary Figures

Supplementary Figure 1

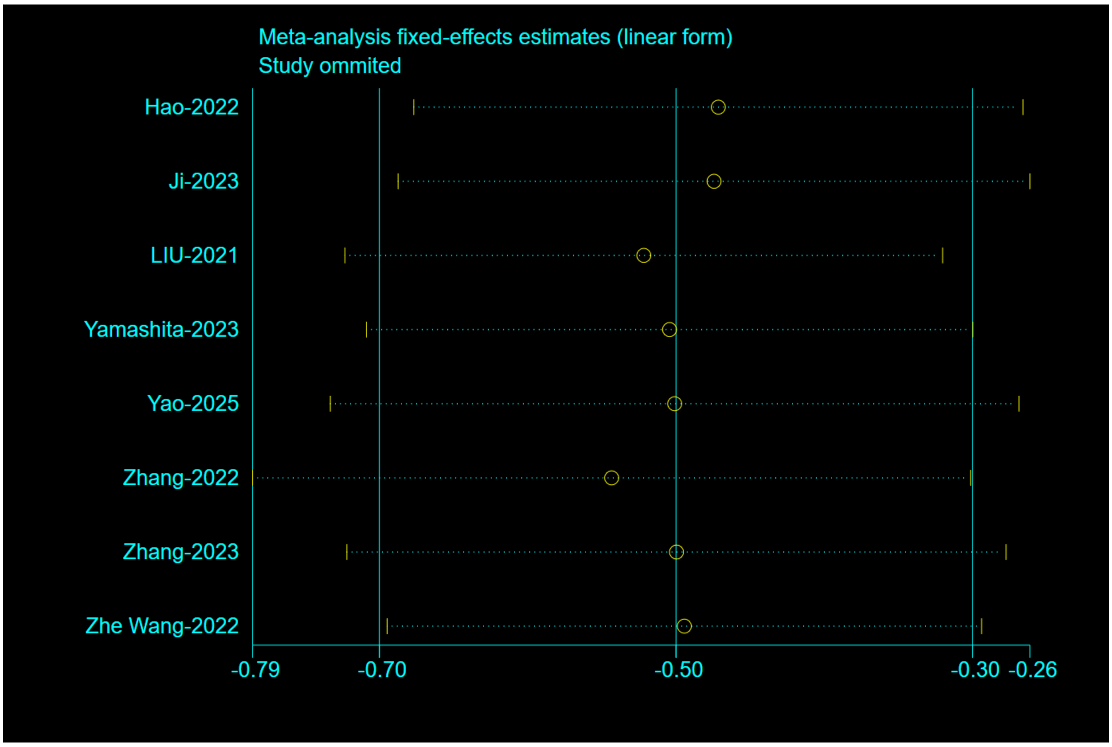

Supplementary Figure 1 Sensitivity analysis for MACE

Supplementary Figure 2

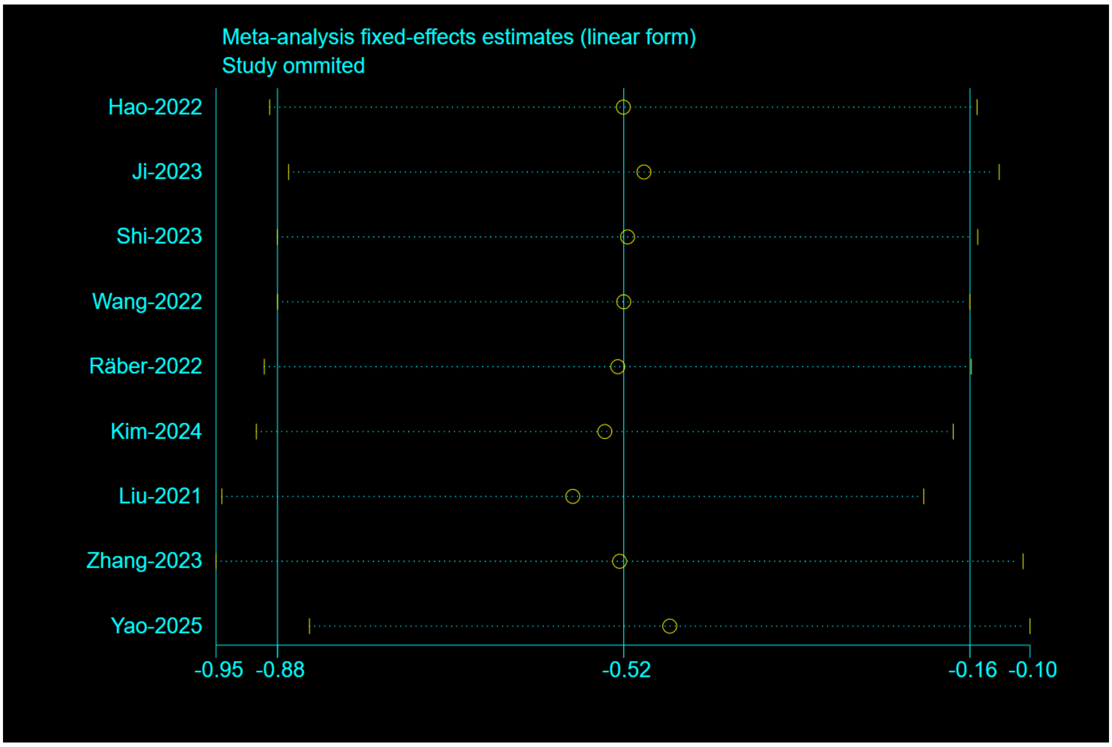

Supplementary Figure 2 Sensitivity analysis for Non-Fatal MI

Supplementary Figure 3

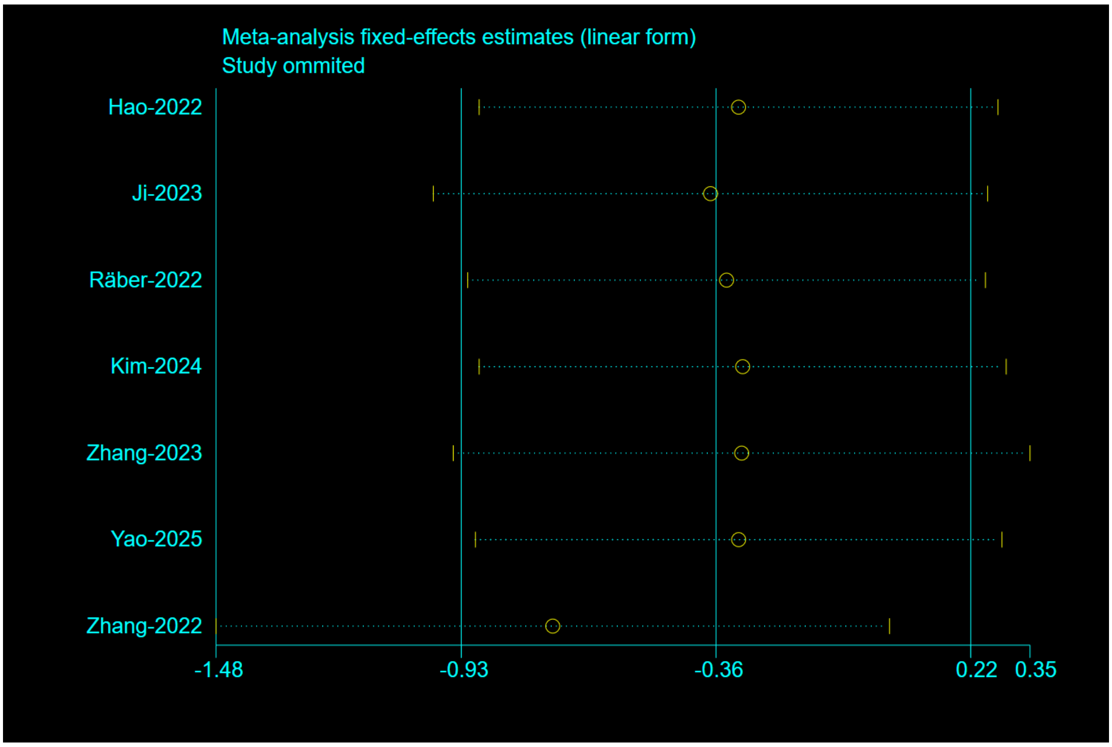

Supplementary Figure 3 Sensitivity analysis for Non-Fatal Stroke

Supplementary Figure 4

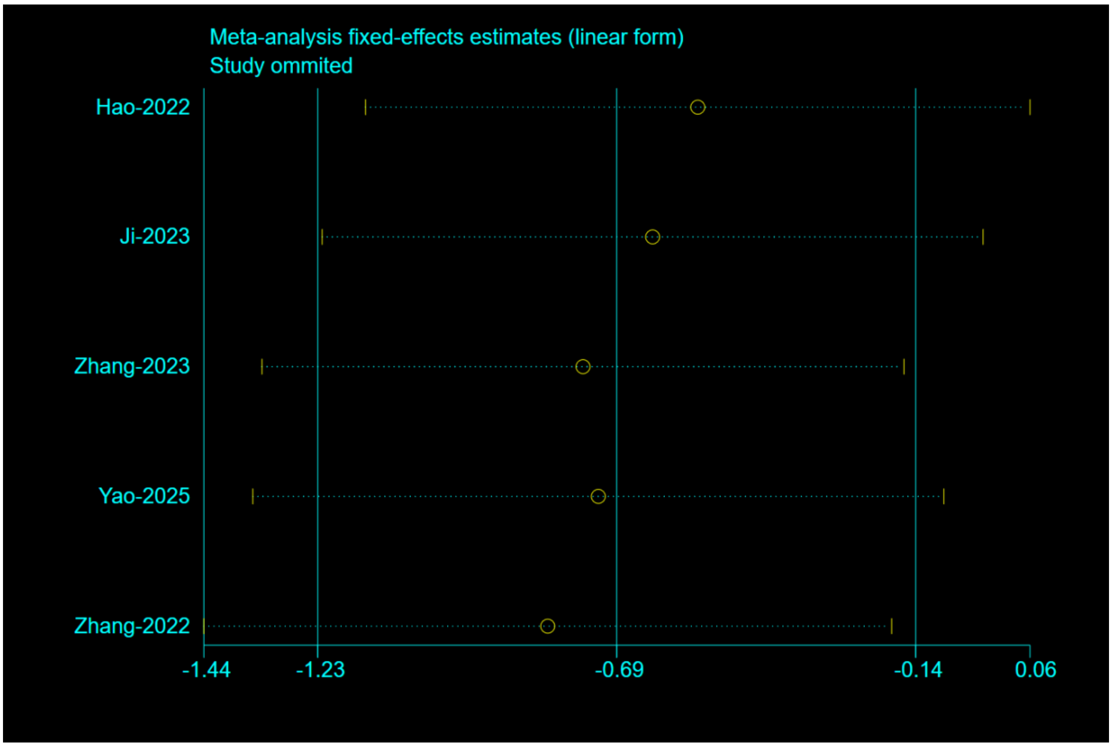

Supplementary Figure 4 Sensitivity analysis for Rehospitalization for Unstable Angina

Supplementary Figure 5

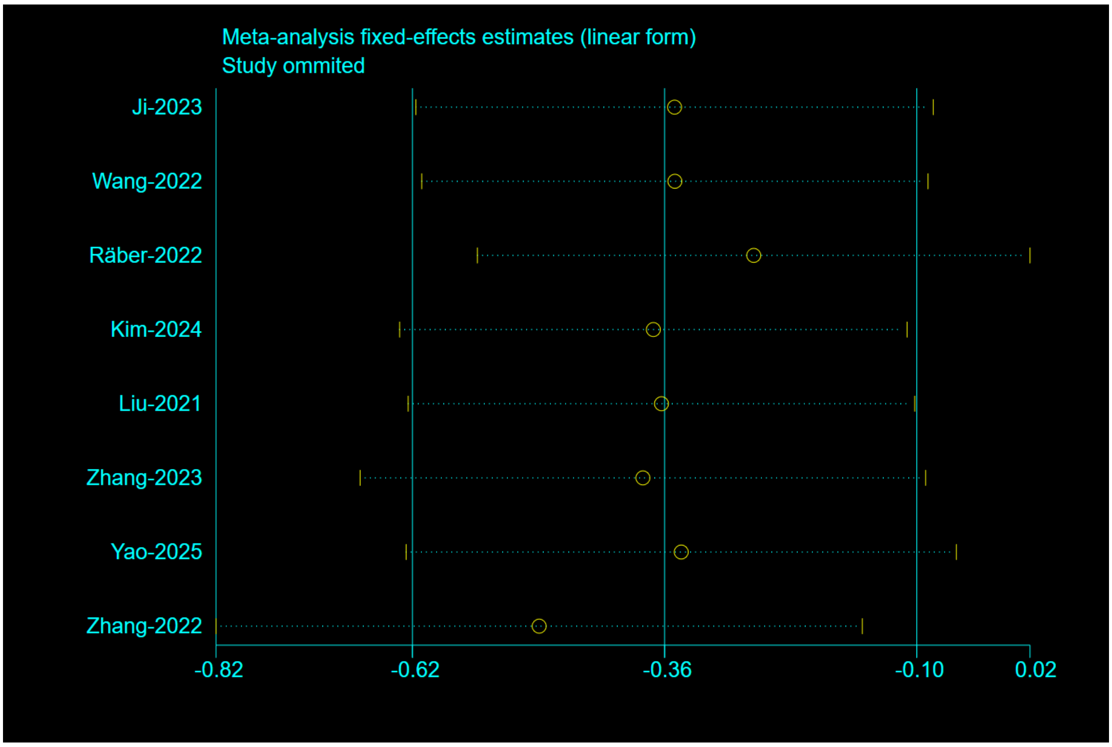

Supplementary Figure 5 Sensitivity analysis for Unplanned Revascularization

Supplementary Figure 6

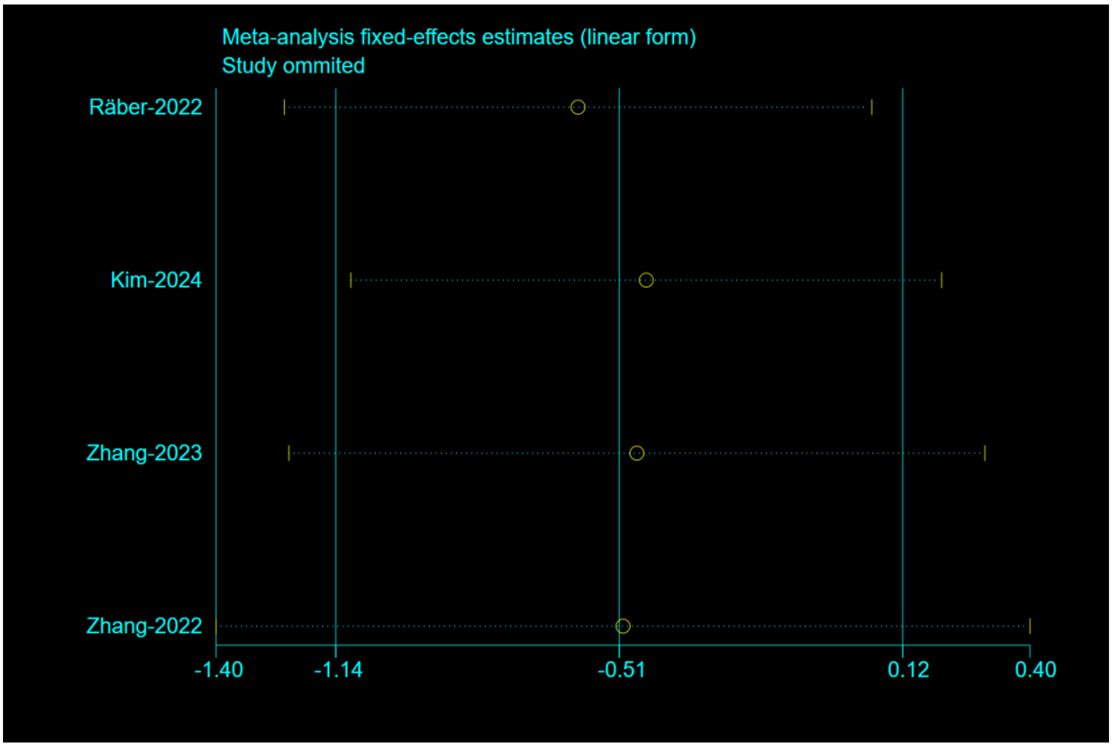

Supplementary Figure 6 Sensitivity analysis for All-Cause Mortality

Supplementary Figure 7

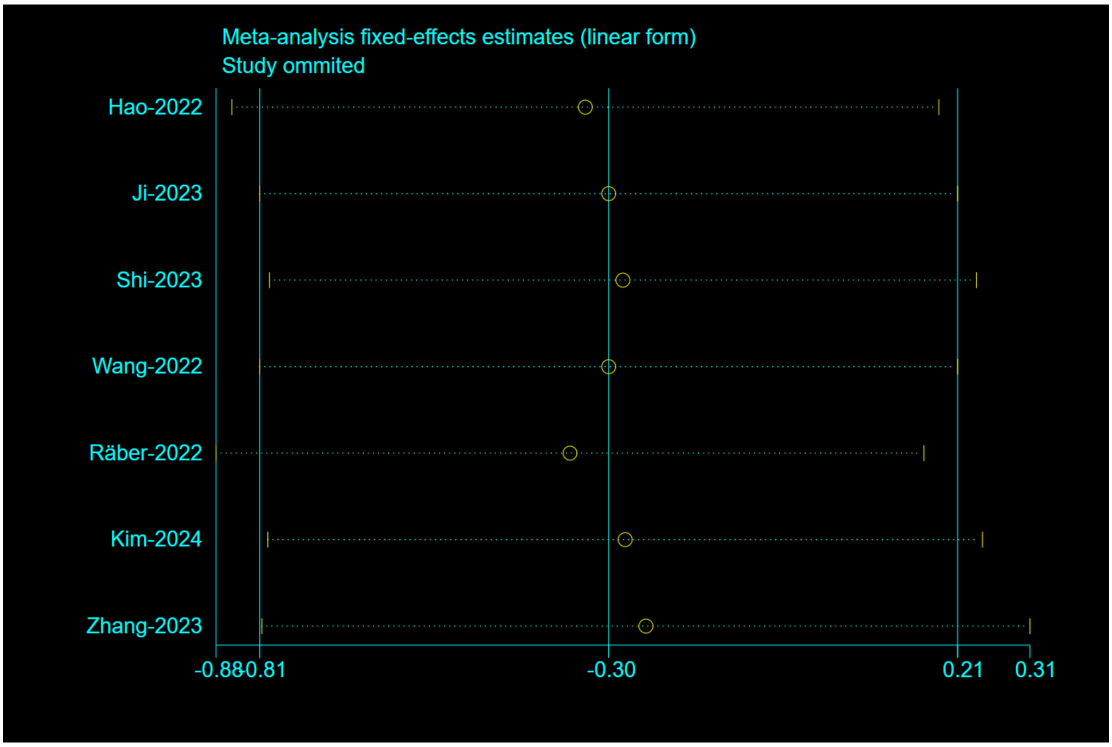

Supplementary Figure 7 Sensitivity analysis for Cardiovascular Mortality

Supplementary Figure 8

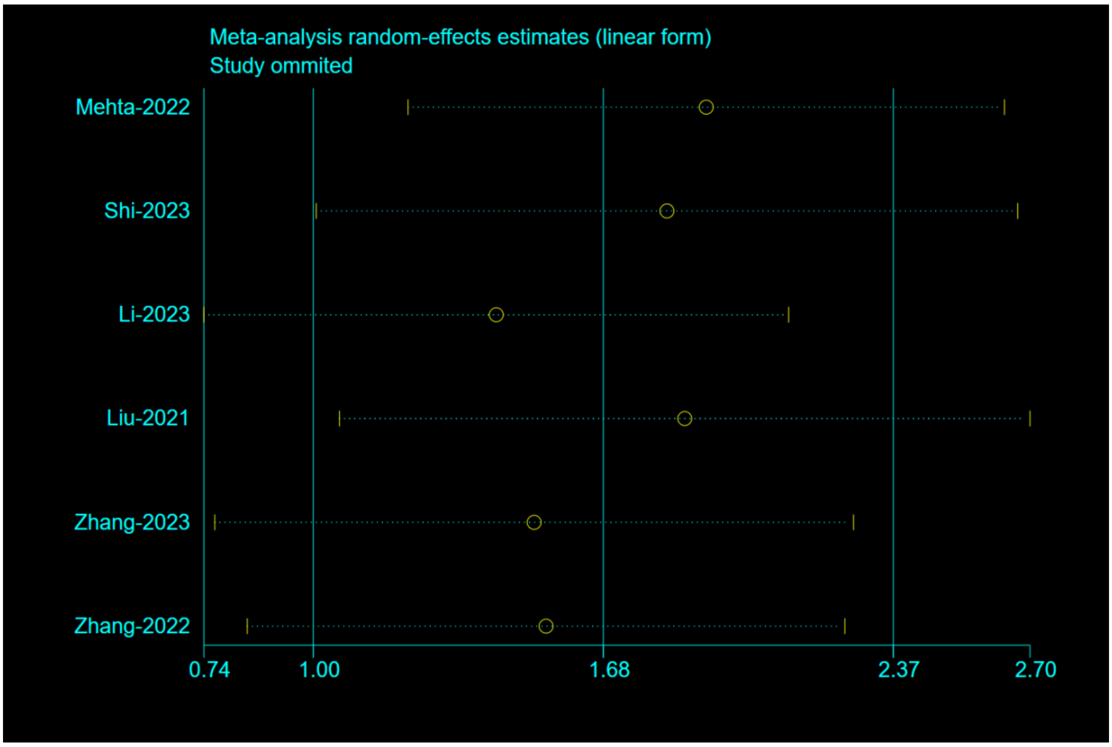

Supplementary Figure 8 Sensitivity analysis for The compliance rate of LDL-C ≤1.4 mmol/L

Supplementary Figure 9

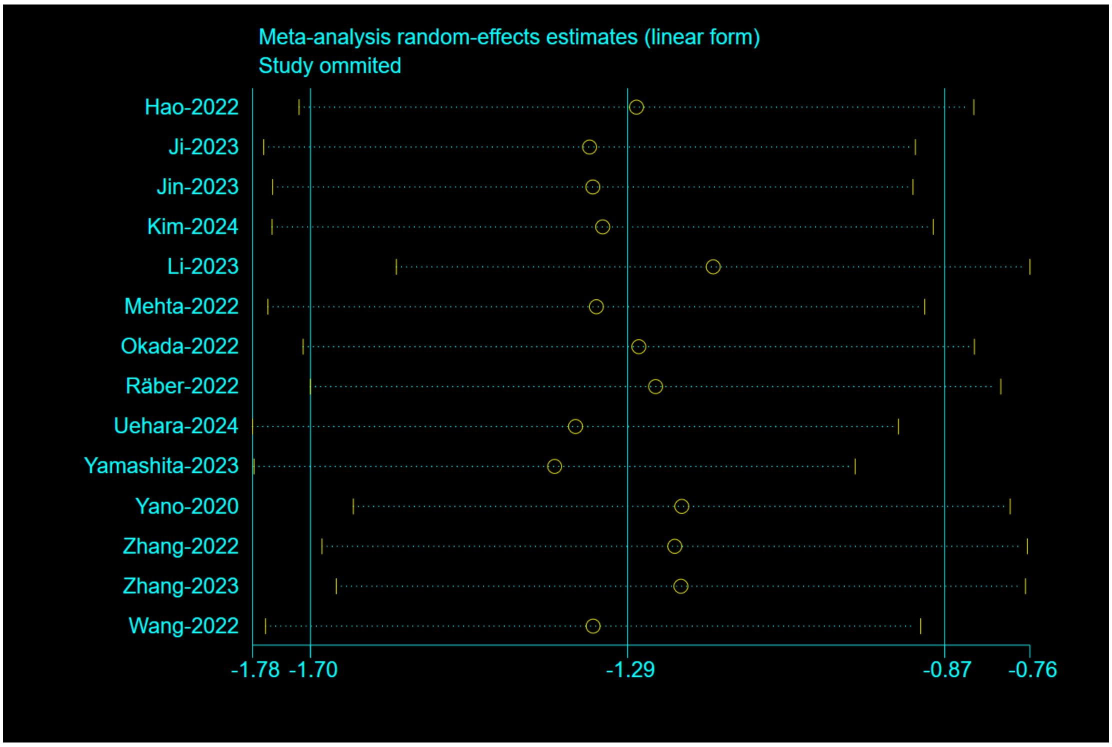

Supplementary Figure 9 Sensitivity analysis for LDL-C

Supplementary Figure 10

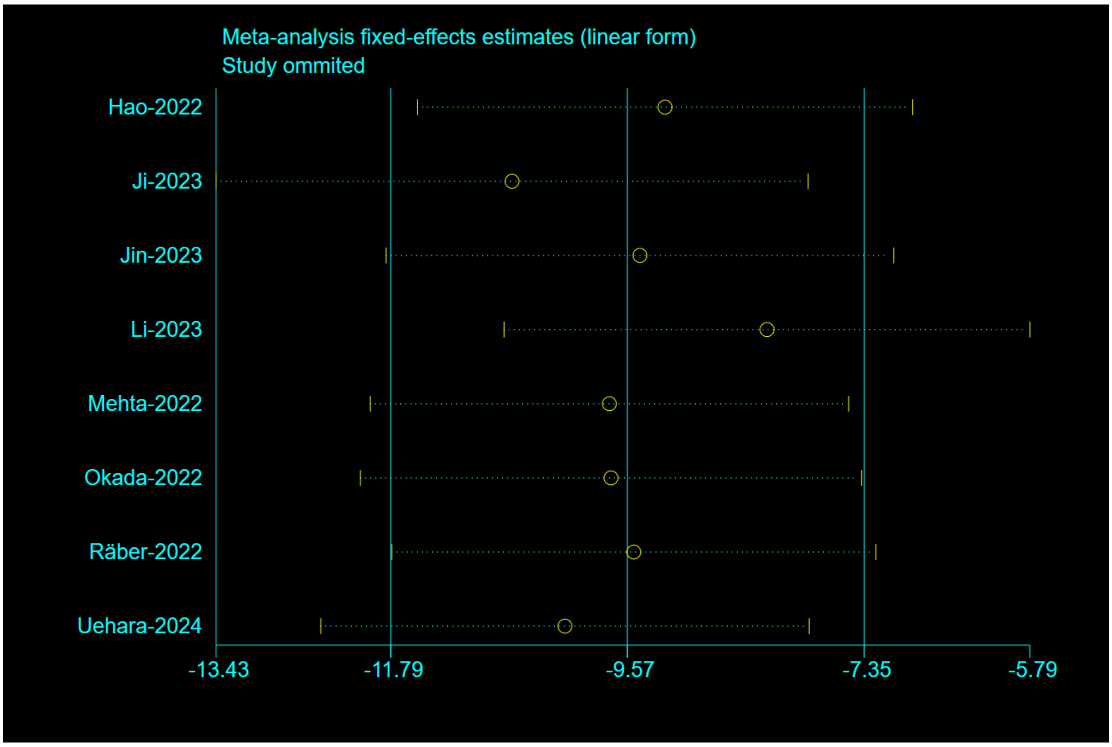

Supplementary Figure 10 Sensitivity analysis for Lp(a)

Supplementary Figure 11

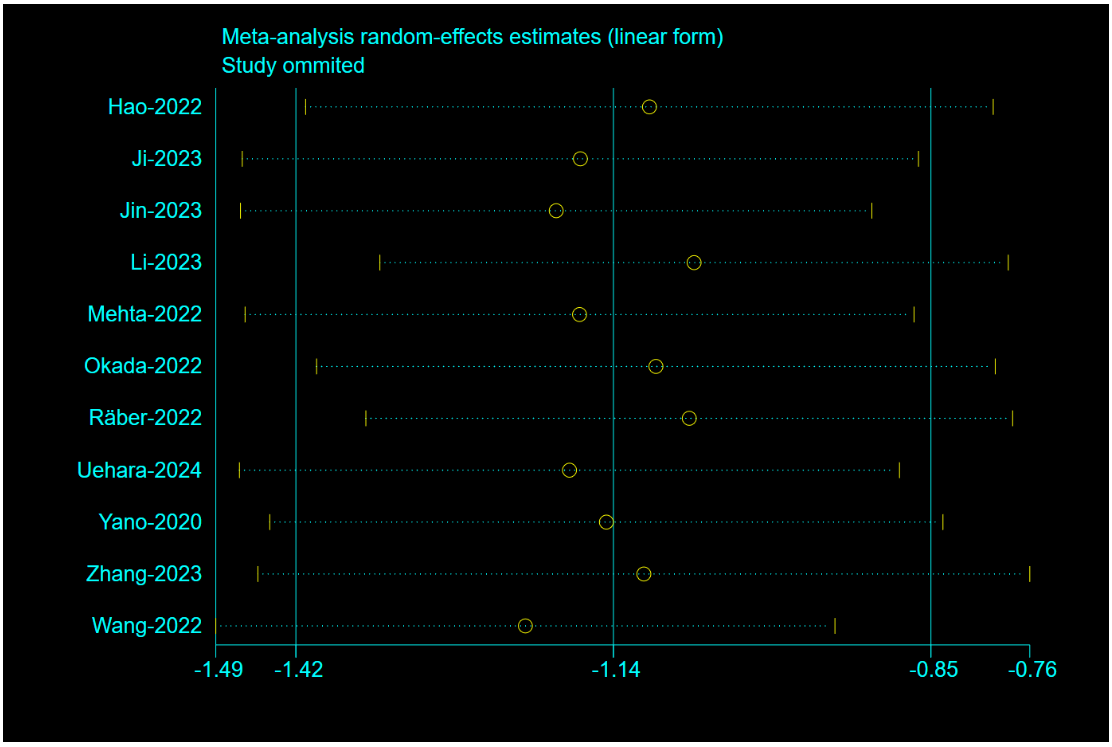

Supplementary Figure 11 Sensitivity analysis for TC

Supplementary Figure 12

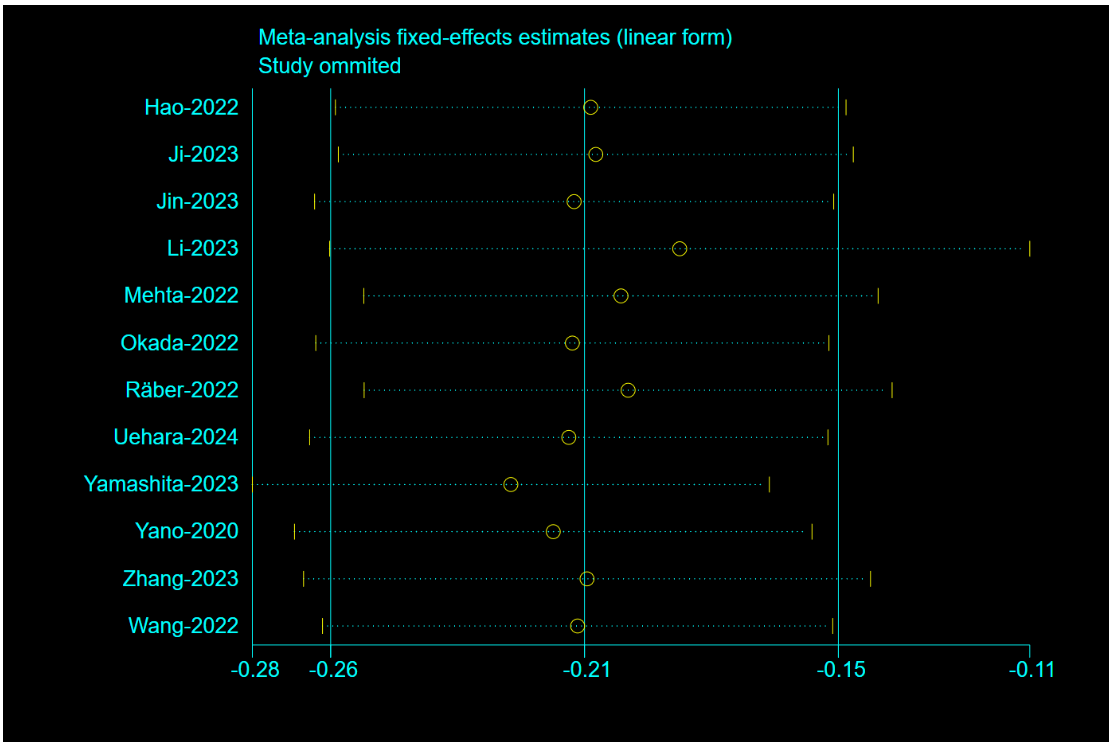

Supplementary Figure 12 Sensitivity analysis for TG

Supplementary Figure 13

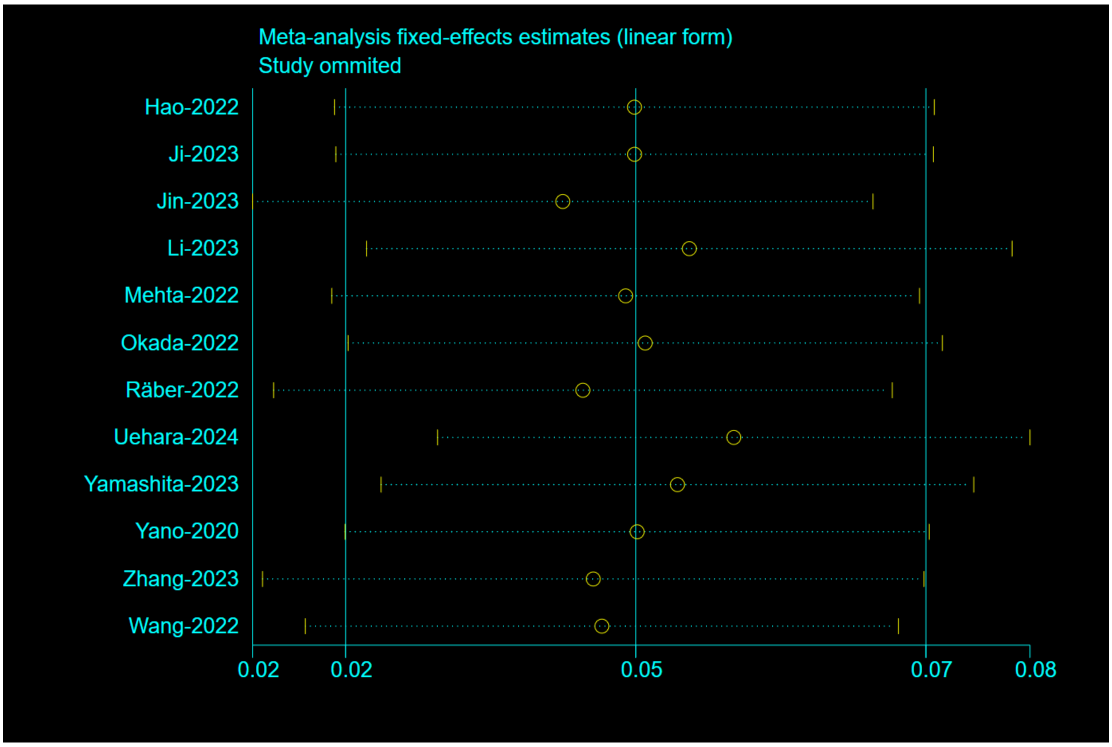

Supplementary Figure 13 Sensitivity analysis for HDL-C

Supplementary Figure 14

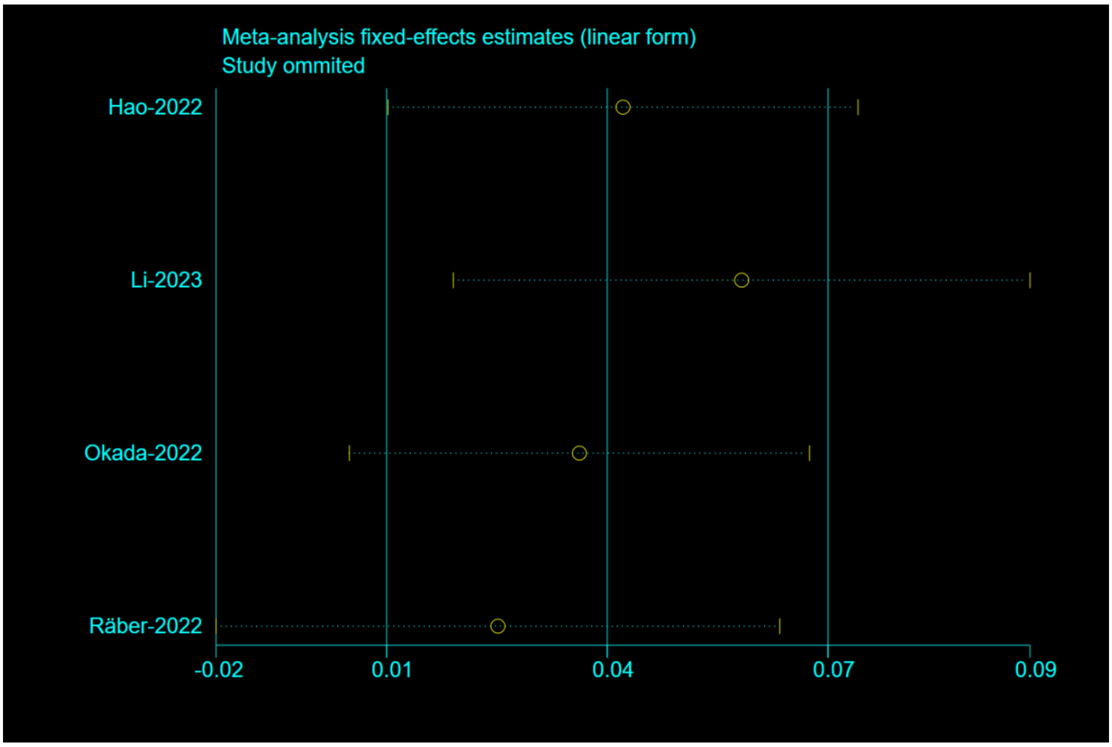

Supplementary Figure 14 Sensitivity analysis for apoA1

Supplementary Figure 15

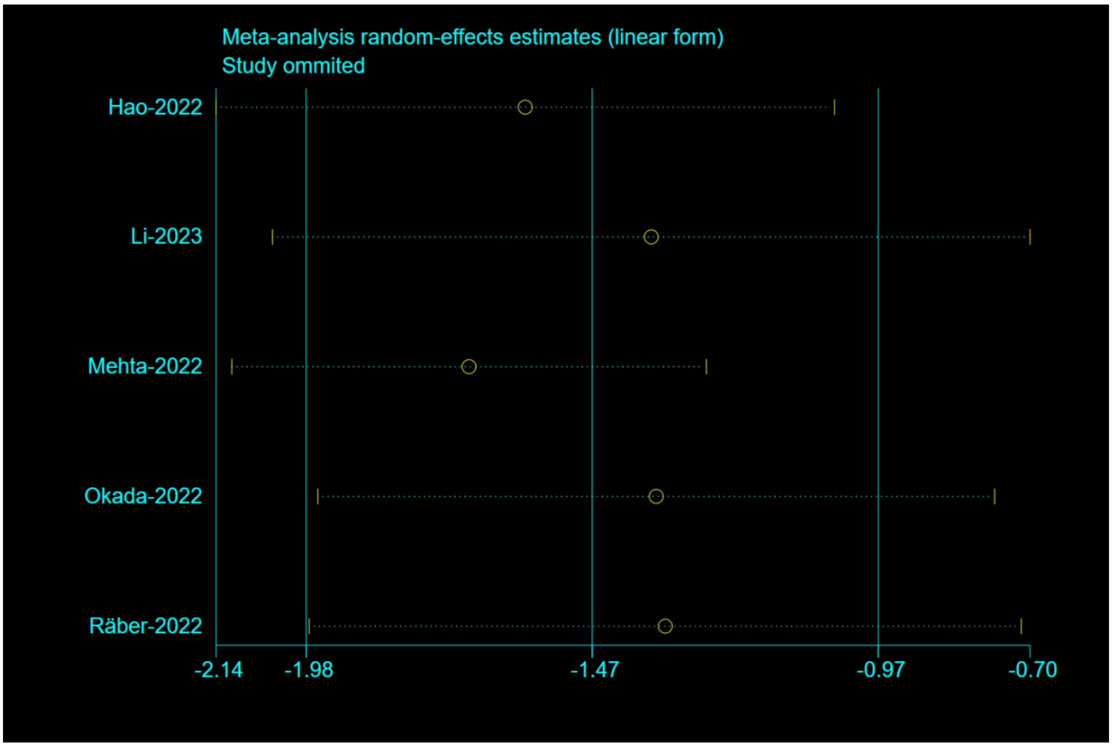

Supplementary Figure 15 Sensitivity analysis for apoB

Supplementary Figure 16

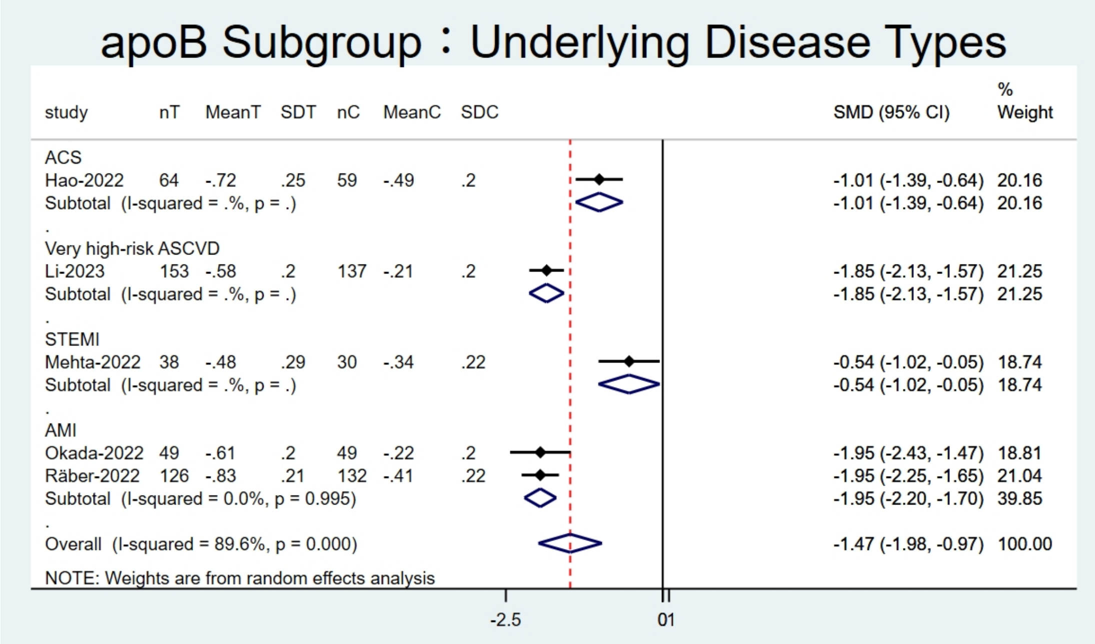

Supplementary Figure 16 Subgroup Analysis of apoB: Underlying Disease Types

Supplementary Figure 17

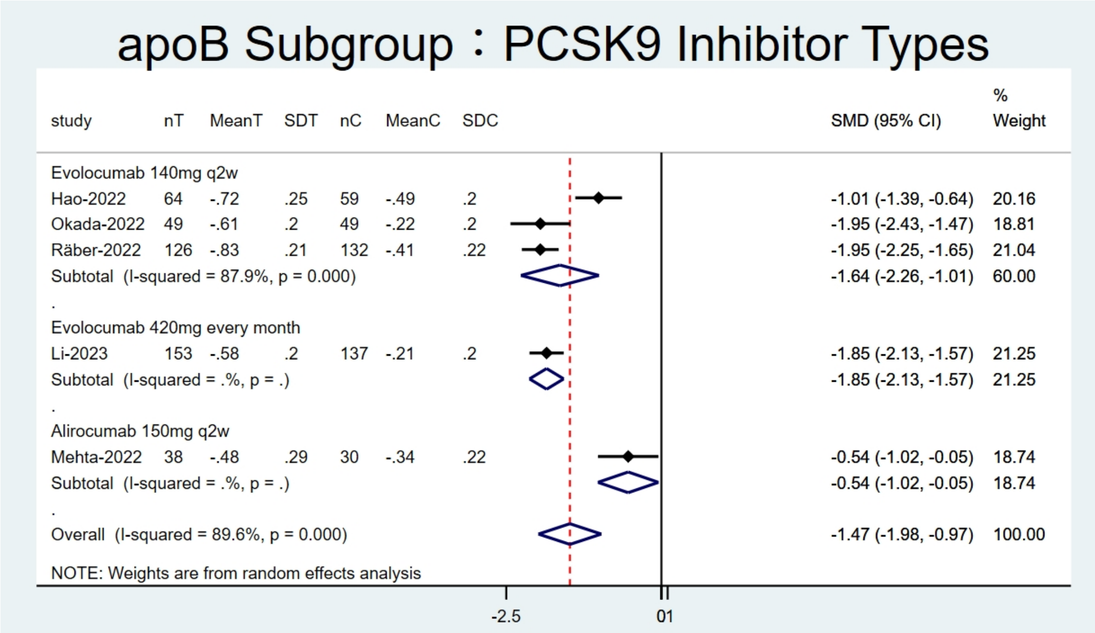

Supplementary Figure 17 Subgroup Analysis of apoB: PCSK9 Inhibitor Types

Supplementary Figure 18

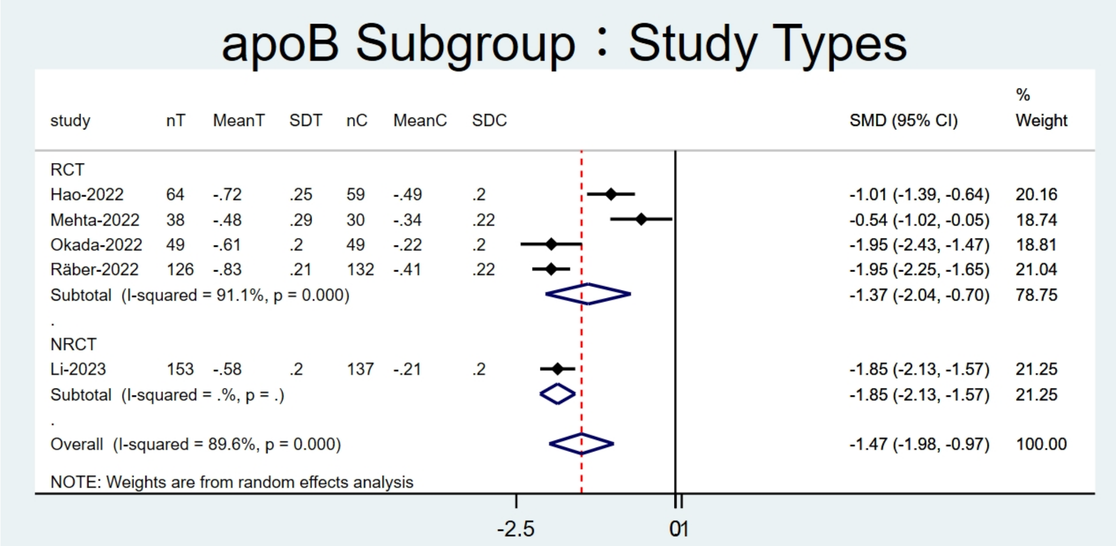

Supplementary Figure 18 Subgroup Analysis of apoB: Study Types

Supplementary Figure 19

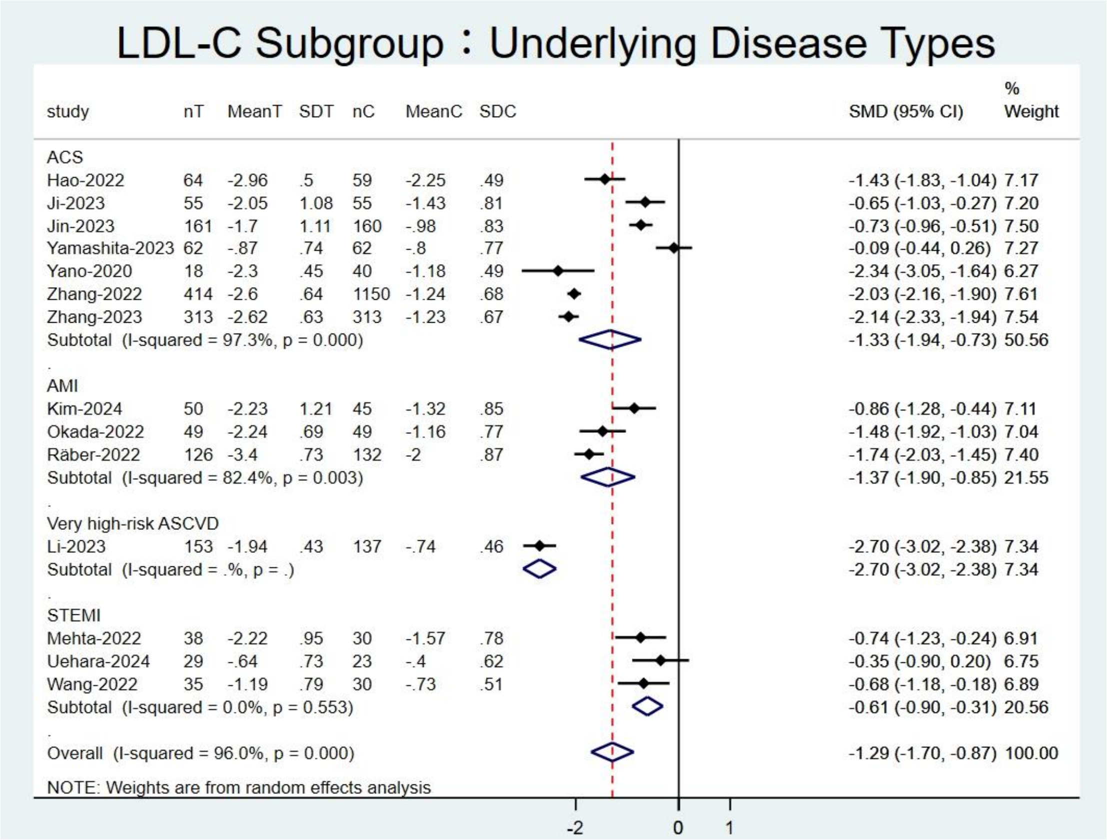

Supplementary Figure 19 Subgroup Analysis of LDL-C: Underlying Disease Types

Supplementary Figure 20

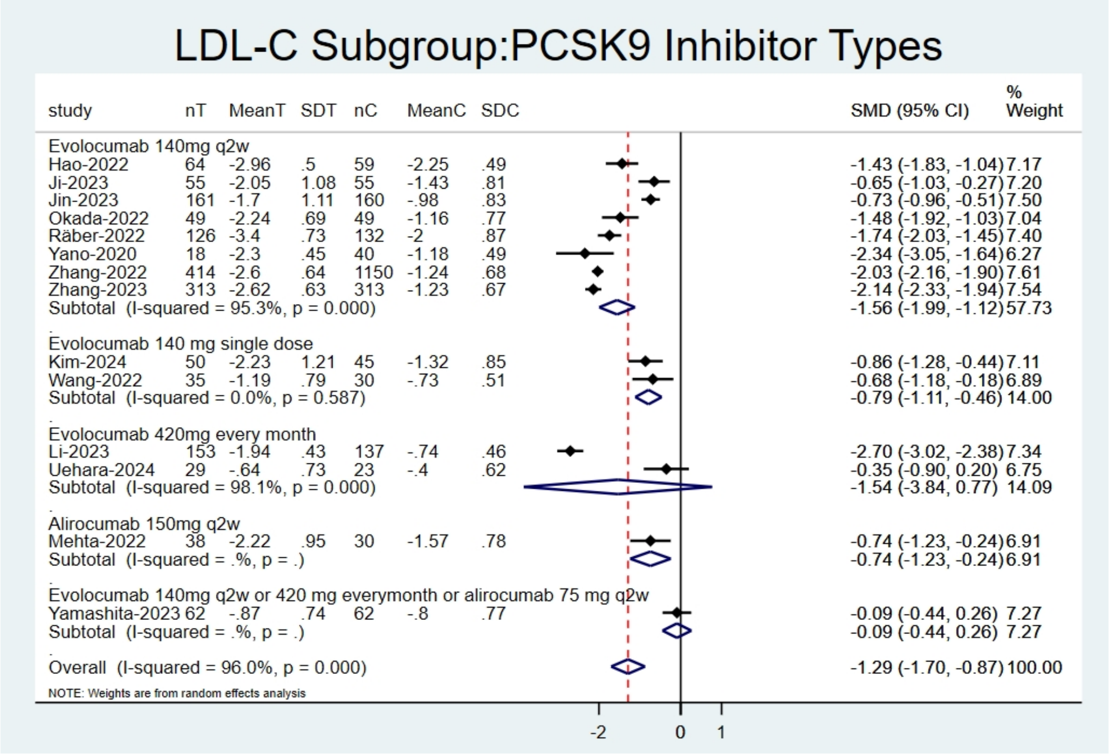

Supplementary Figure 20 Subgroup Analysis of LDL-C: PCSK9 Inhibitor Types

Supplementary Figure 21

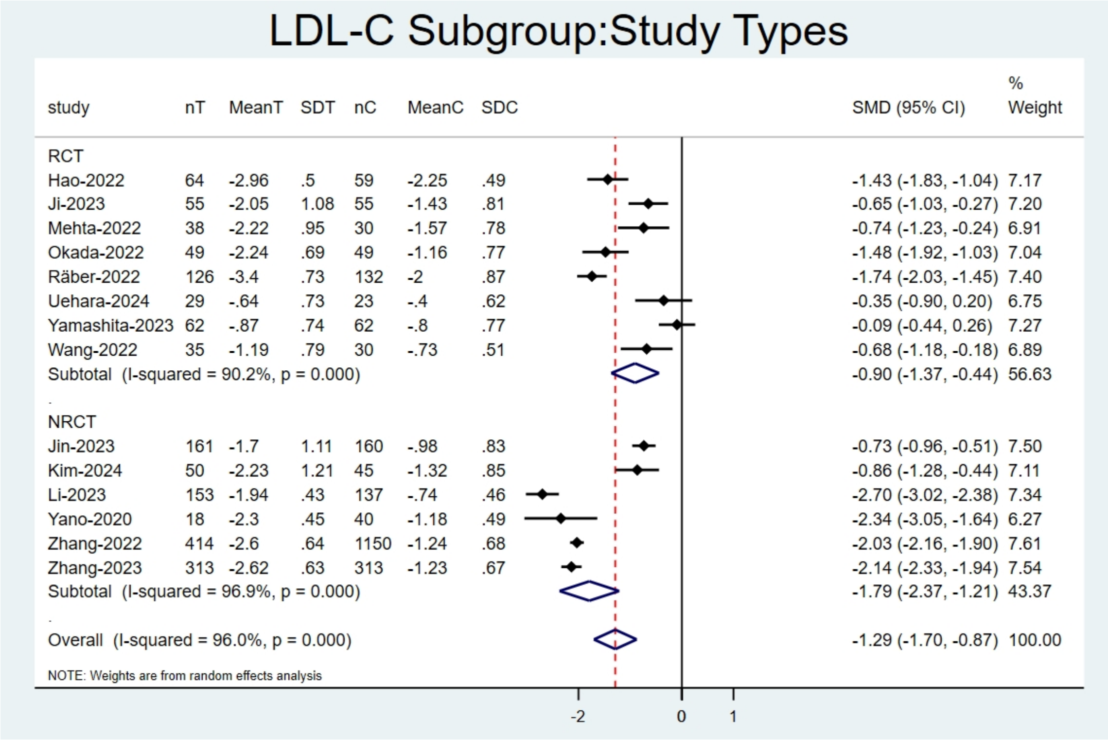

Supplementary Figure 21 Subgroup Analysis of LDL-C: Study Types

Supplementary Figure 22

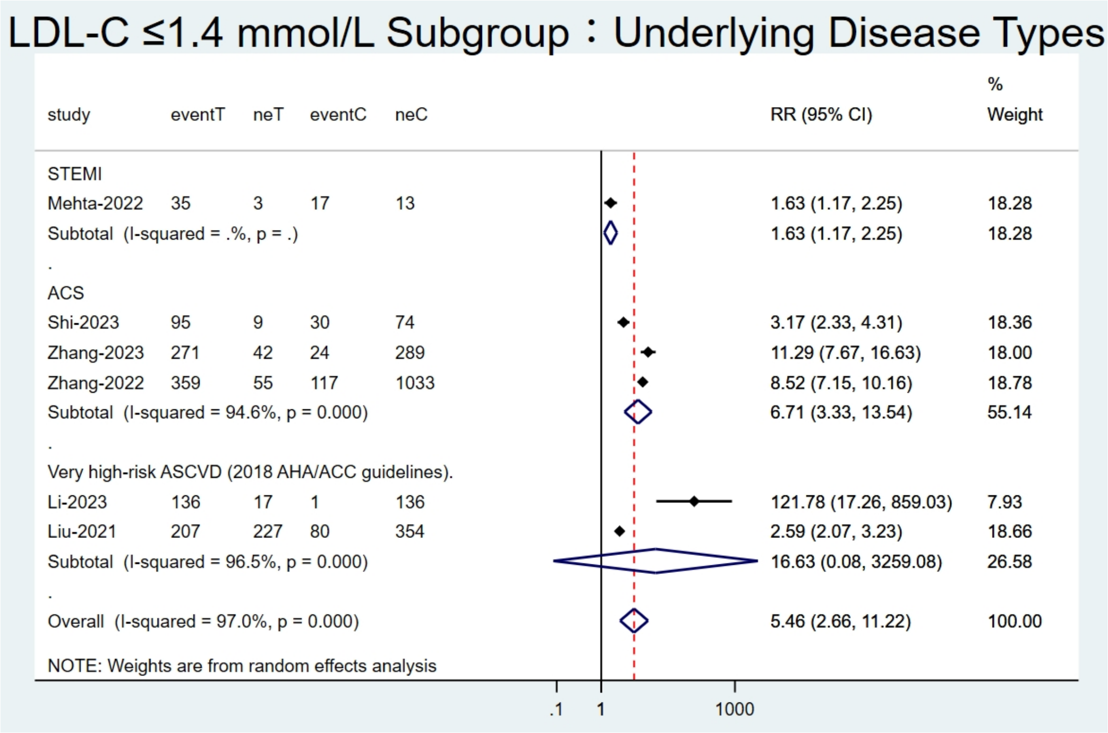

Supplementary Figure 22 Subgroup Analysis of The compliance rate of LDL-C ≤1.4 mmol/L: Underlying Disease Types

Supplementary Figure 23

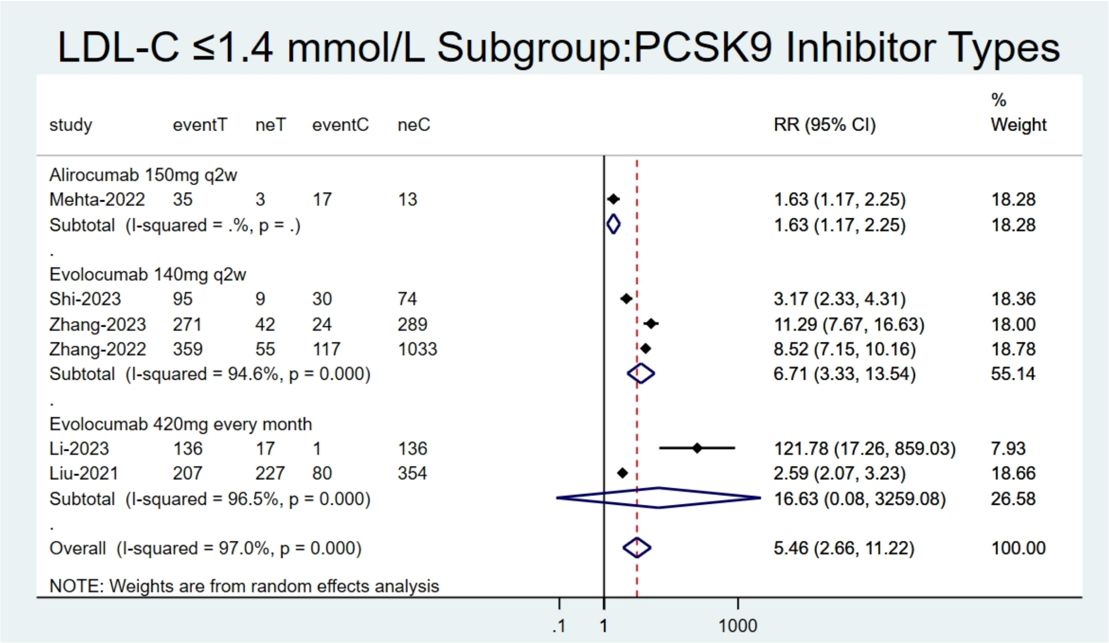

Supplementary Figure 23 Subgroup Analysis of The compliance rate of LDL-C ≤1.4 mmol/L: PCSK9 Inhibitor Types

Supplementary Figure 24

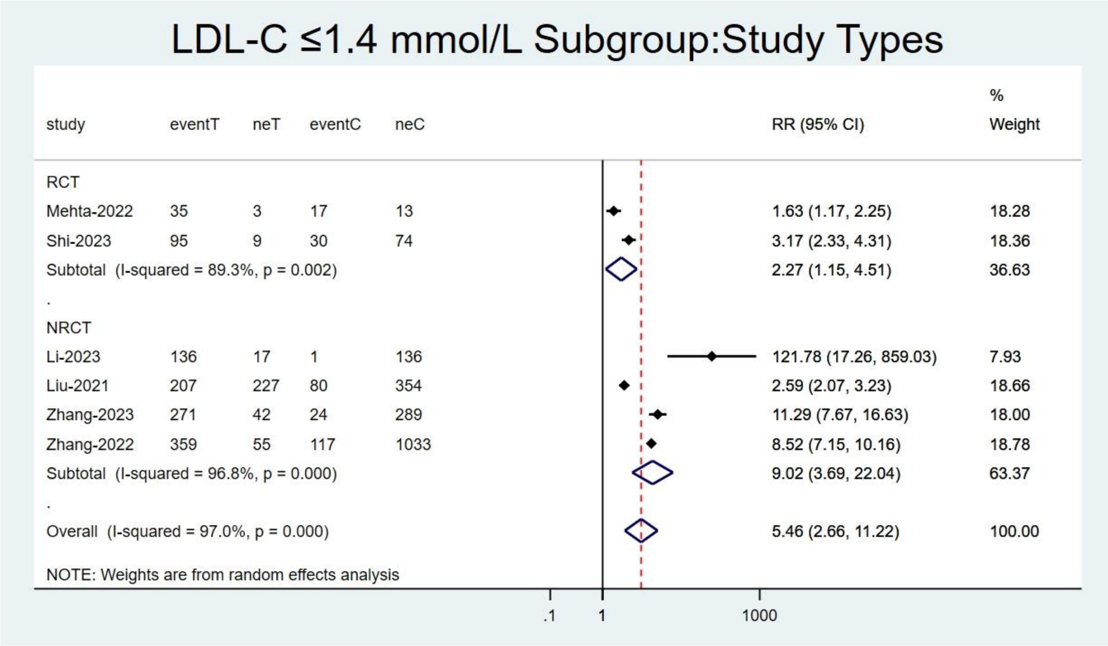

Supplementary Figure 24 Subgroup Analysis of The compliance rate of LDL-C ≤1.4 mmol/L: Study Types

Supplementary Figure 25

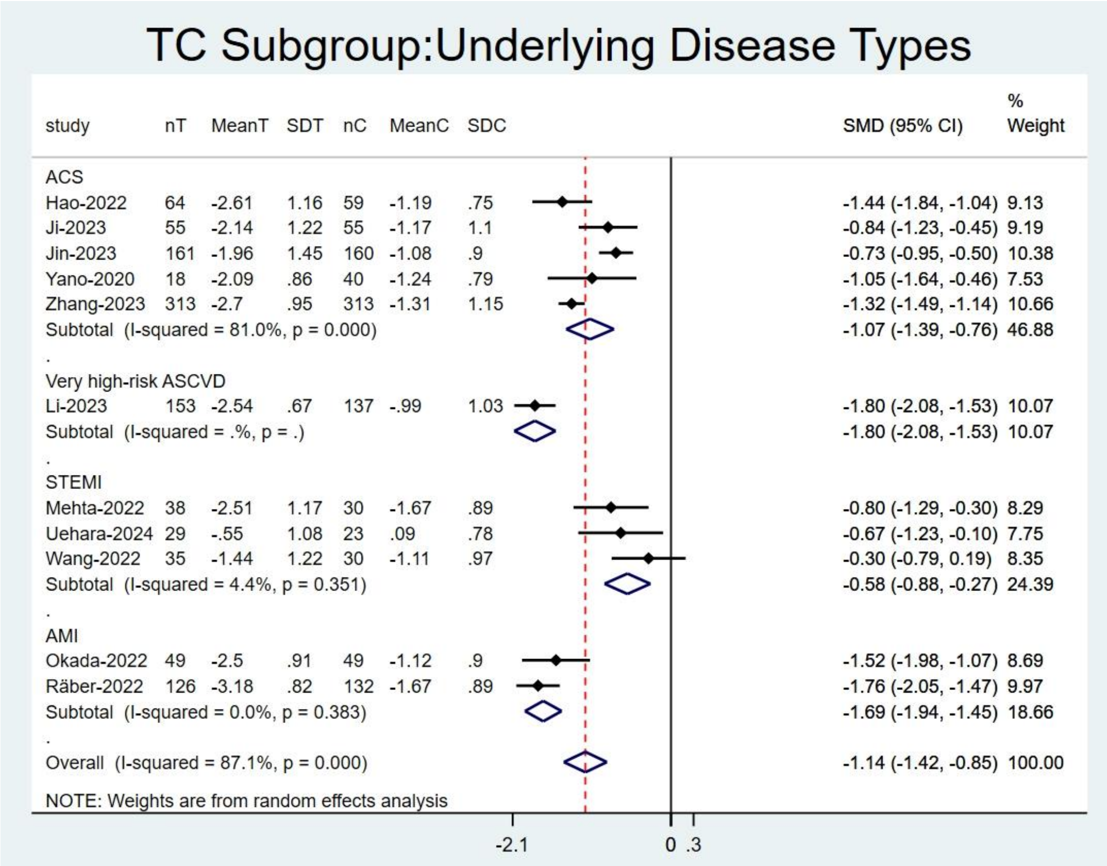

Supplementary Figure 25 Subgroup Analysis of TC: Underlying Disease Types

Supplementary Figure 26

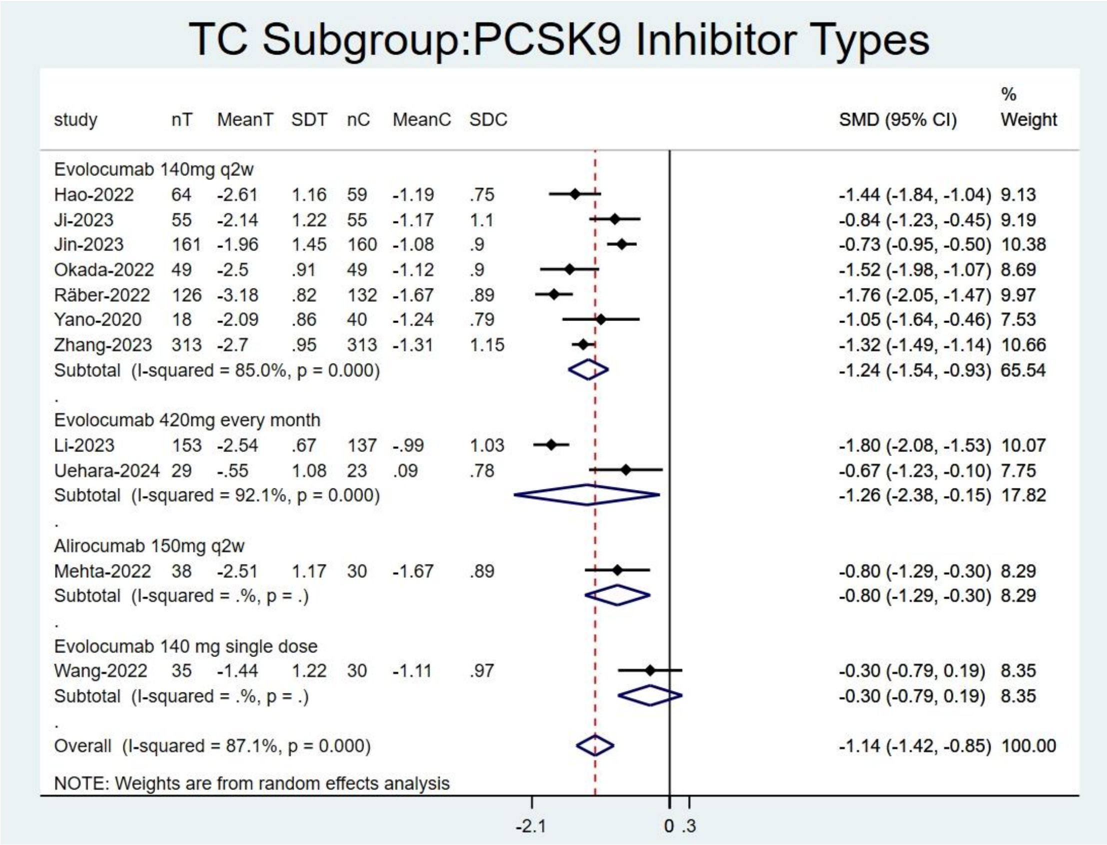

Supplementary Figure 26 Subgroup Analysis of TC: PCSK9 Inhibitor Types

Supplementary Figure 27

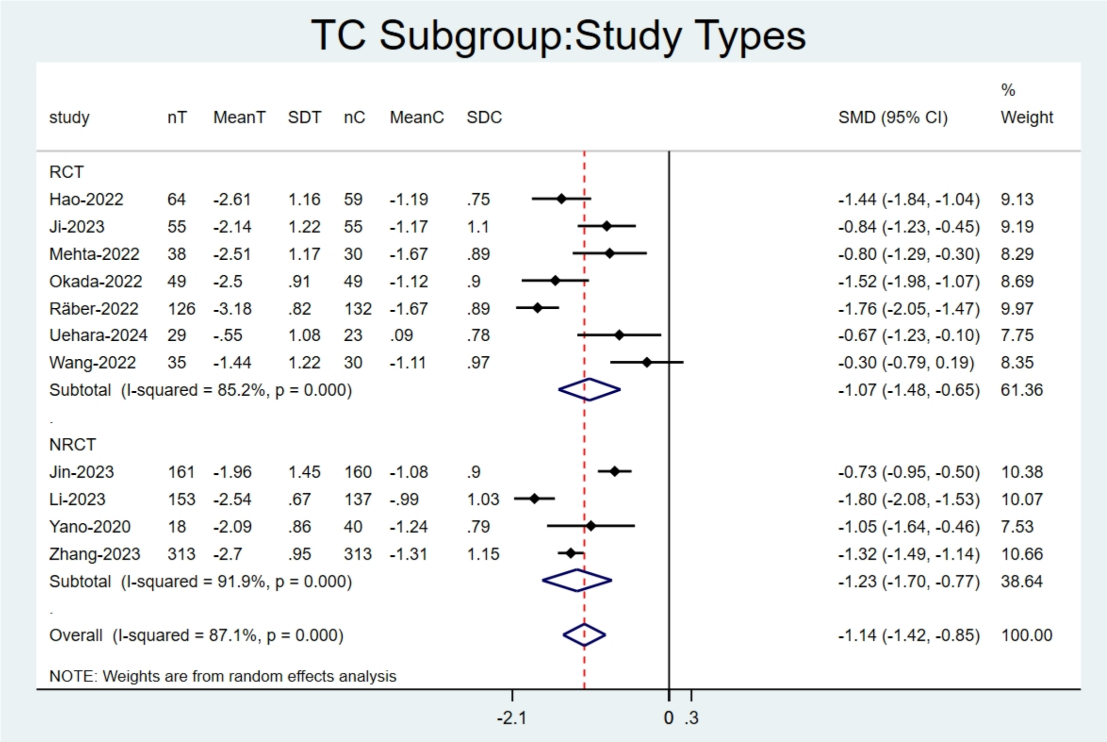

Supplementary Figure 27 Subgroup Analysis of TC: Study Types

Supplementary Figure 28

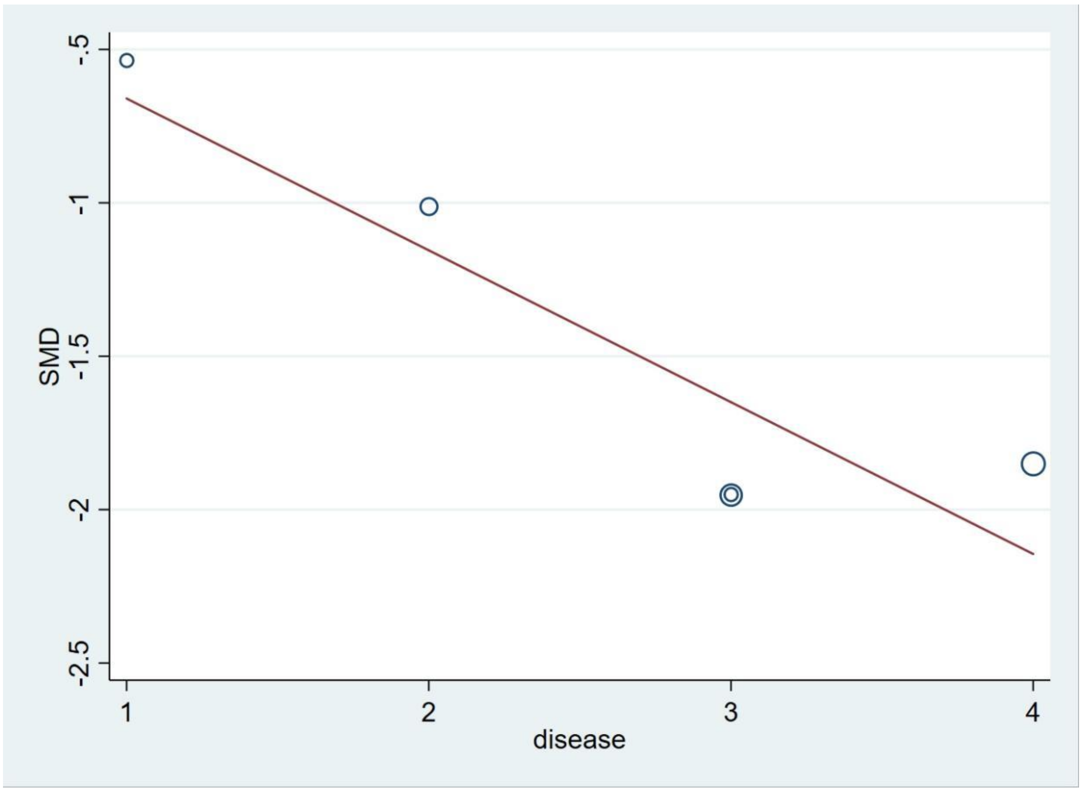

Supplementary Figure 28 Meta-regression analysis of apoB: Underlying Disease Types

Supplementary Figure 29

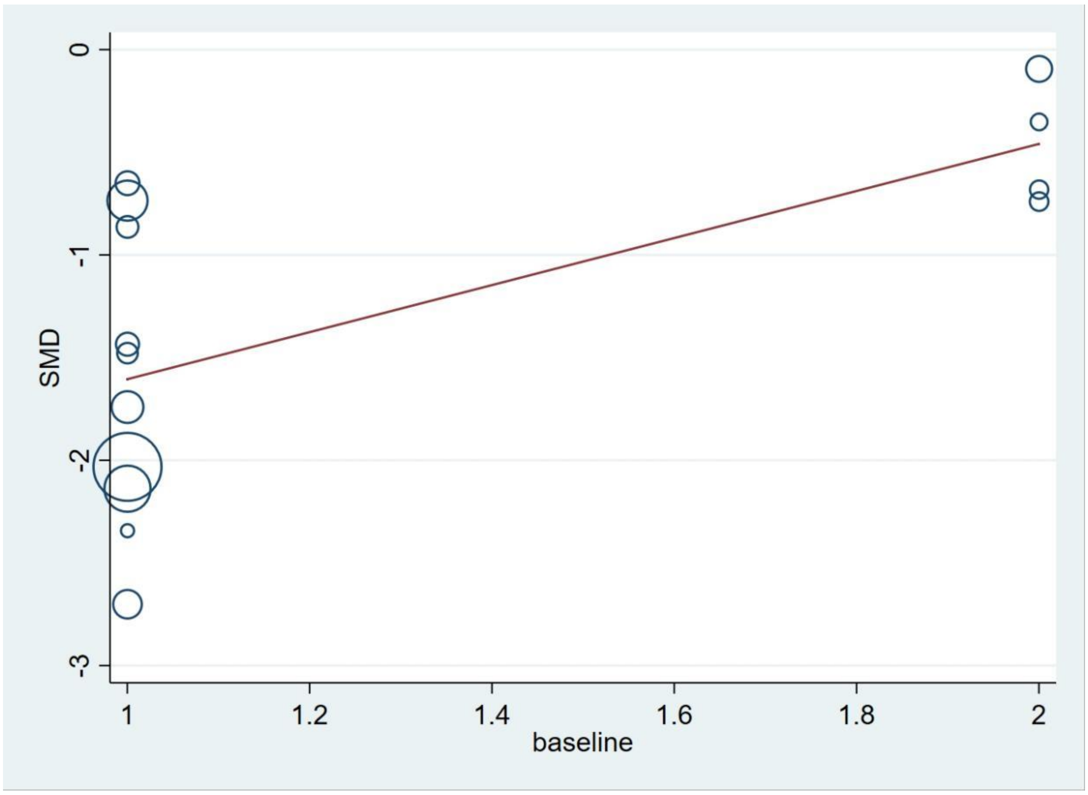

Supplementary Figure 29 Meta-regression analysis of LDL-C: Baseline LDL-C levels(> 3mmol/L or ≤3mmol/L)

Supplementary Figure 30

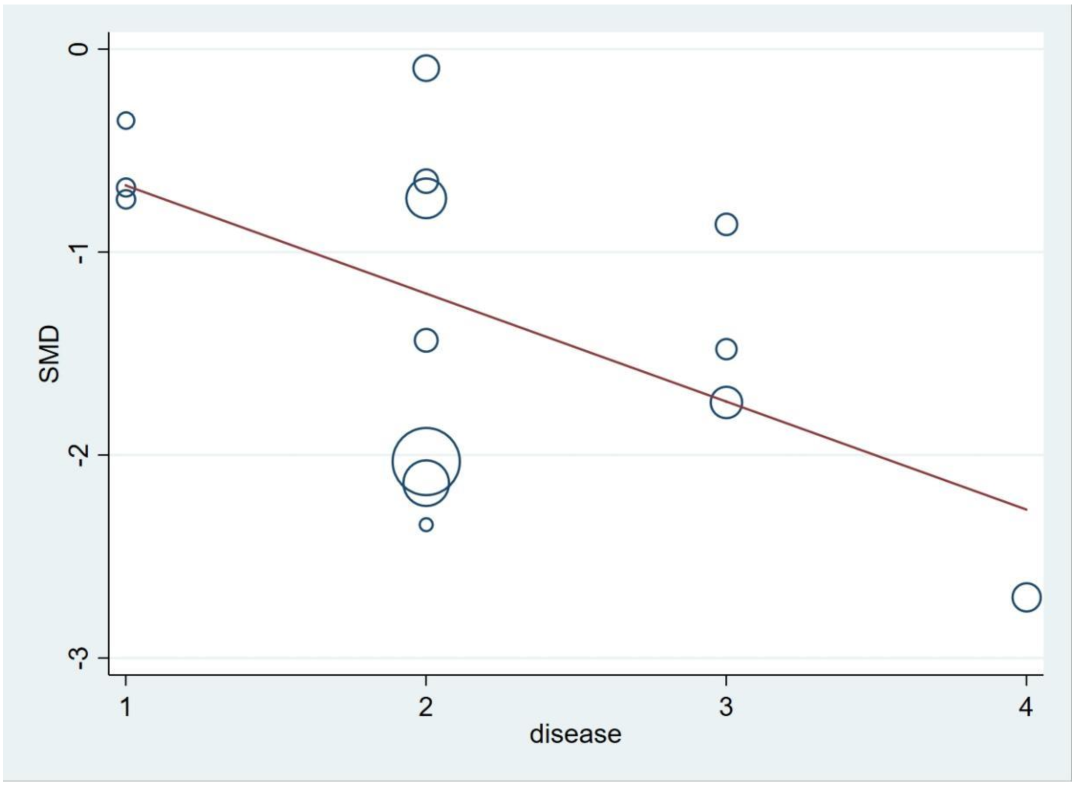

Supplementary Figure 30 Meta-regression analysis of LDL-C: Underlying Disease Types

Supplementary Figure 31

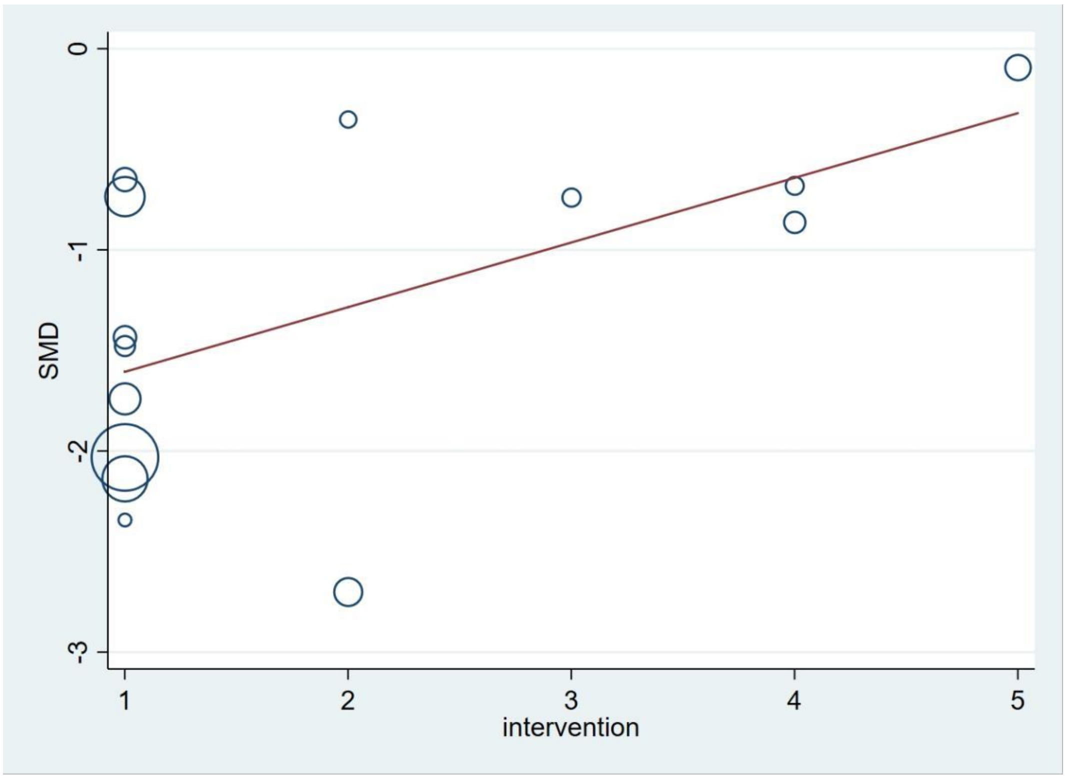

Supplementary Figure 31 Meta-regression analysis of LDL-C: PCSK9 Inhibitor Types

Supplementary Figure 32

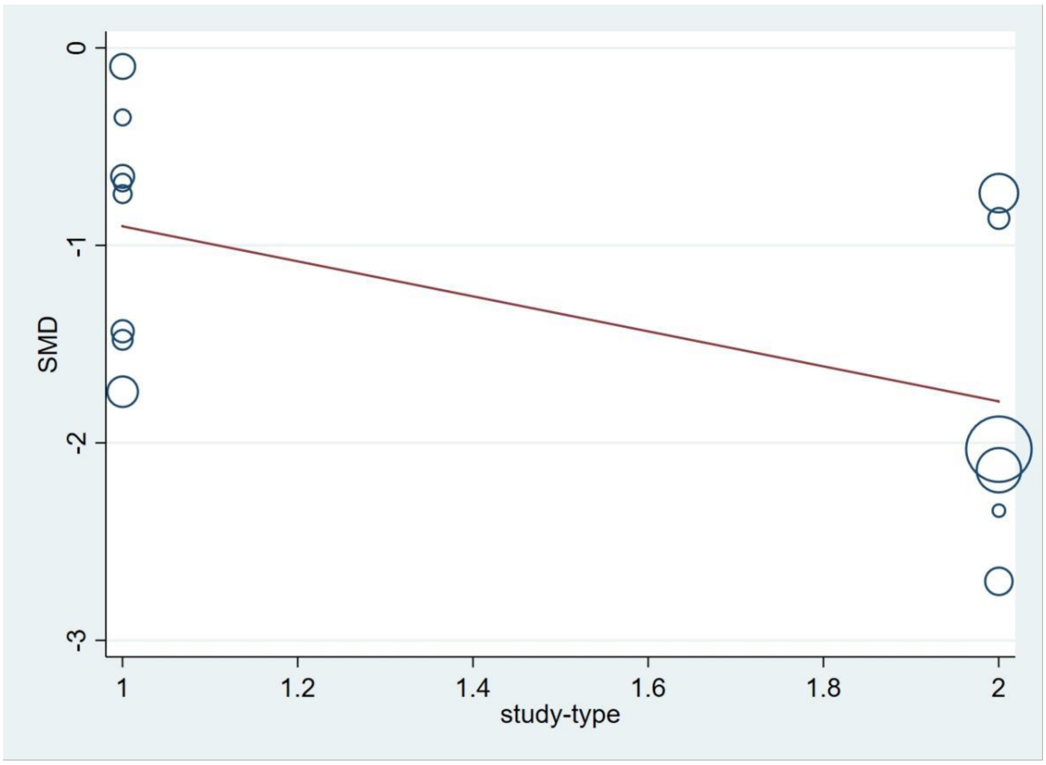

Supplementary Figure 32 Meta-regression analysis of LDL-C: Study Types

Supplementary Figure 33

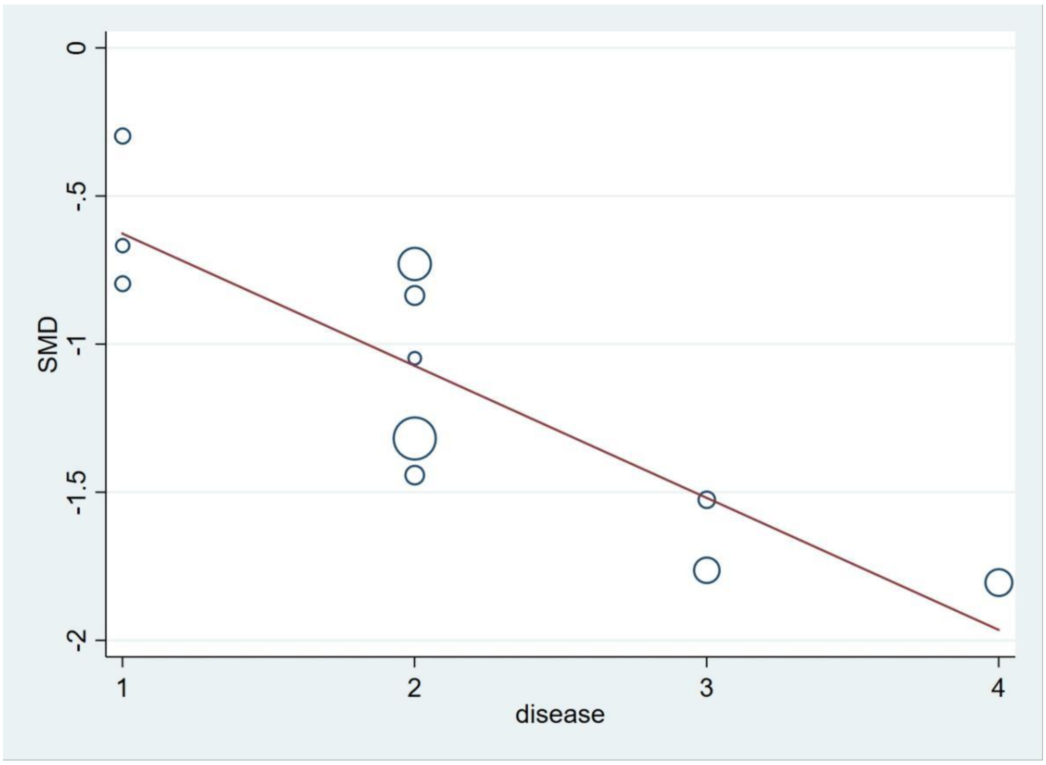

Supplementary Figure 33 Meta-regression analysis of TC: Underlying Disease Types

Supplementary Figure 34

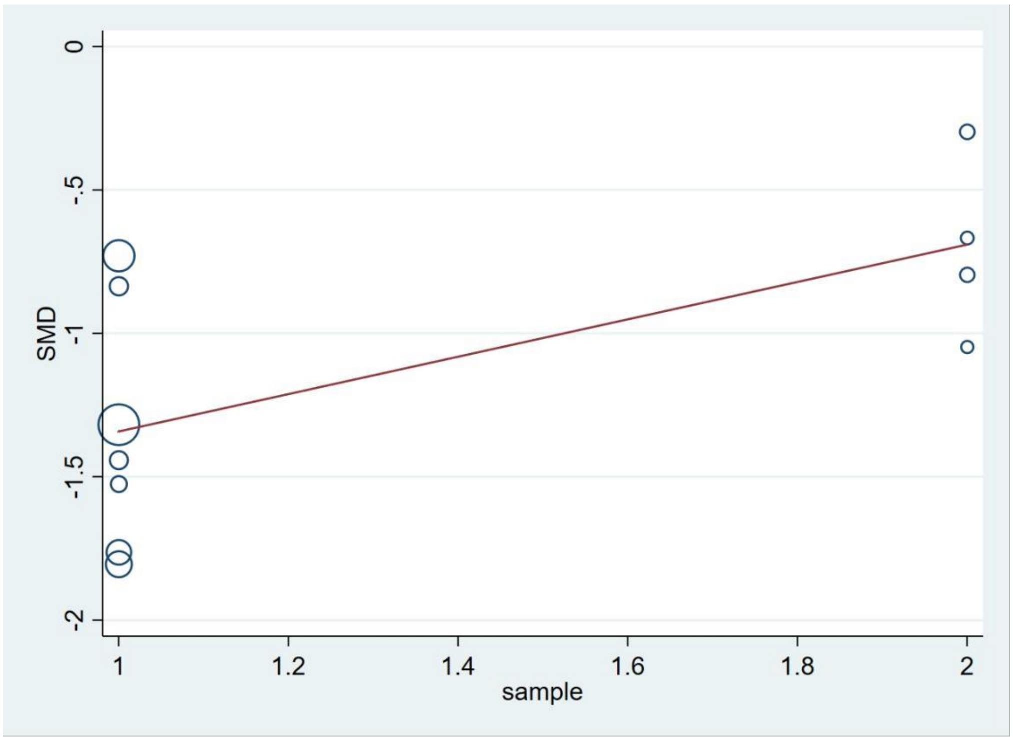

Supplementary Figure 34 Meta-regression analysis of TC: Sample Size(> 100 or ≤100)

Supplementary Figure 35

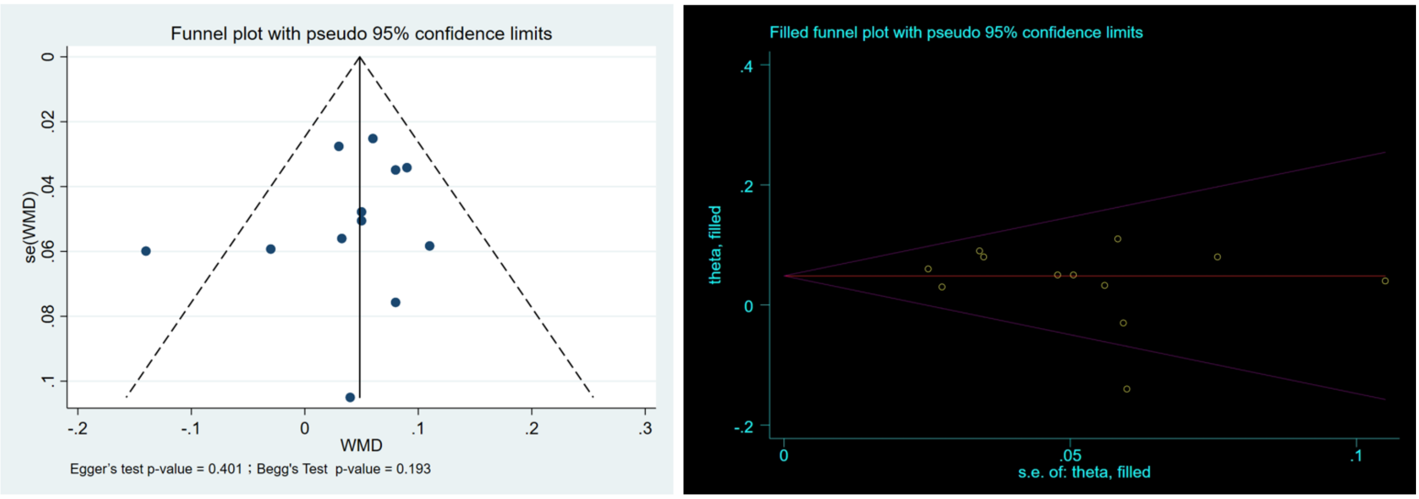

Supplementary Figure 35 Funnel Plot and Trim and Fill Plot of HDL-C

Supplementary Figure 36

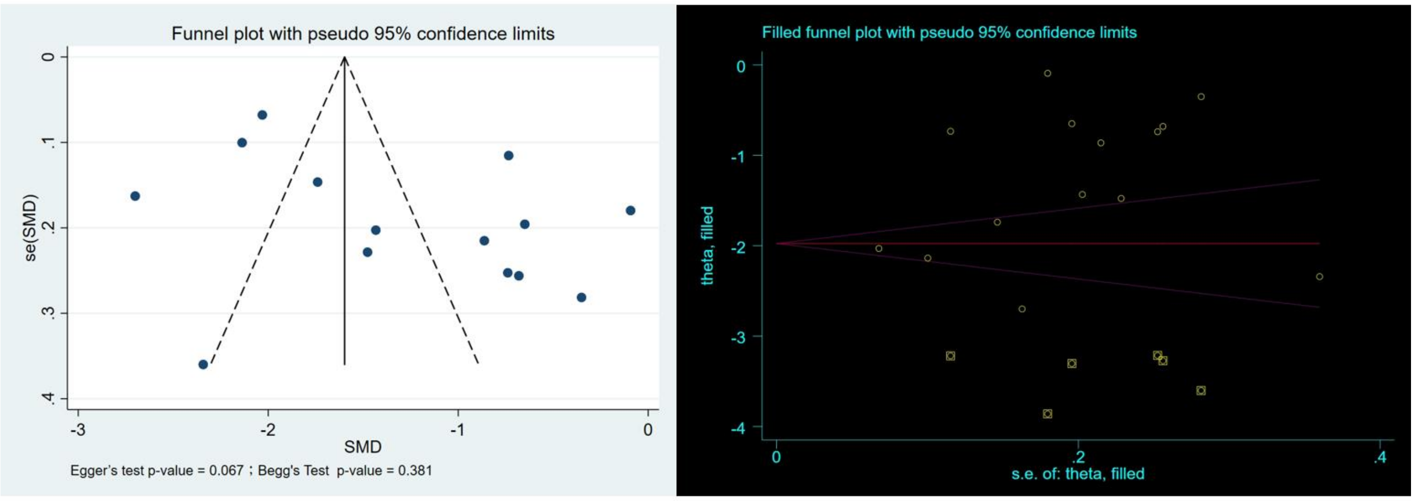

Supplementary Figure 36 Funnel Plot and Trim and Fill Plot of LDL-C

Supplementary Figure 37

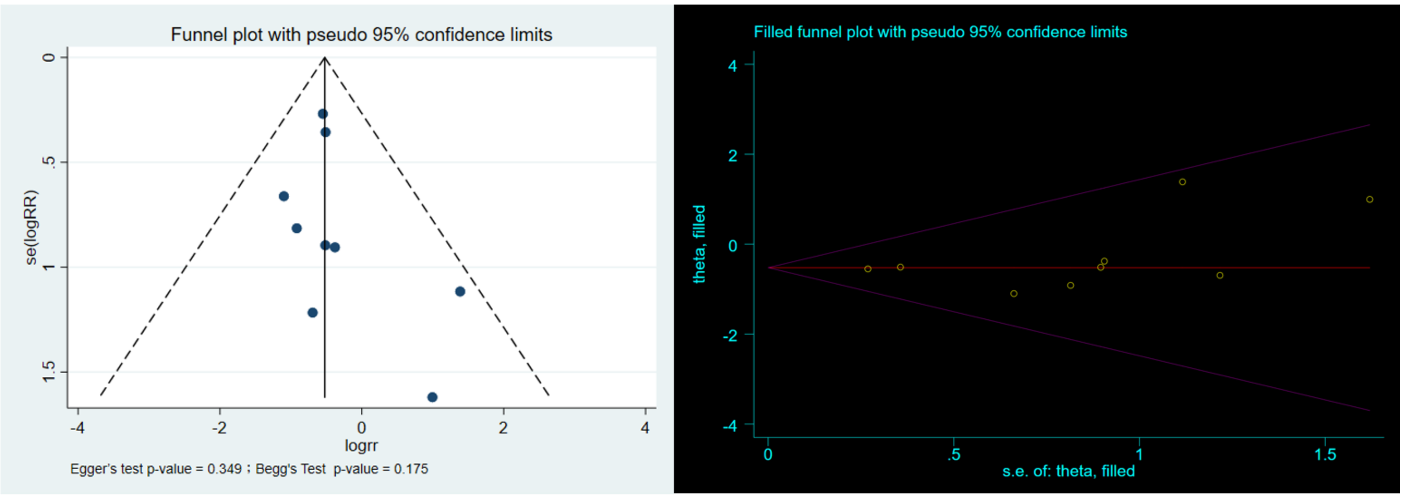

Supplementary Figure 37 Funnel Plot and Trim and Fill Plot of Non-Fatal MI

Supplementary Figure 38

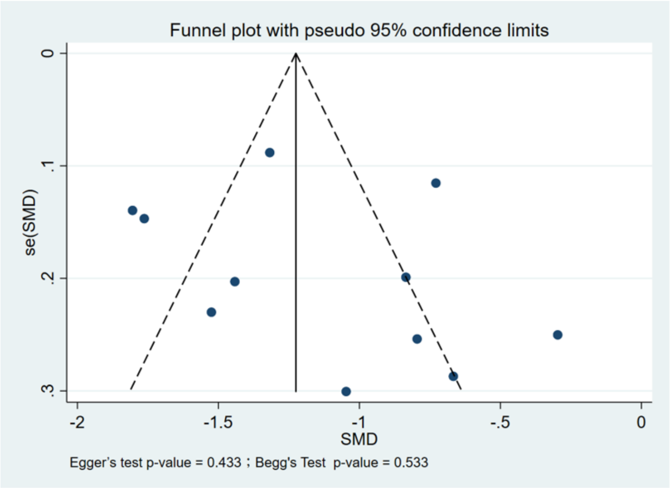

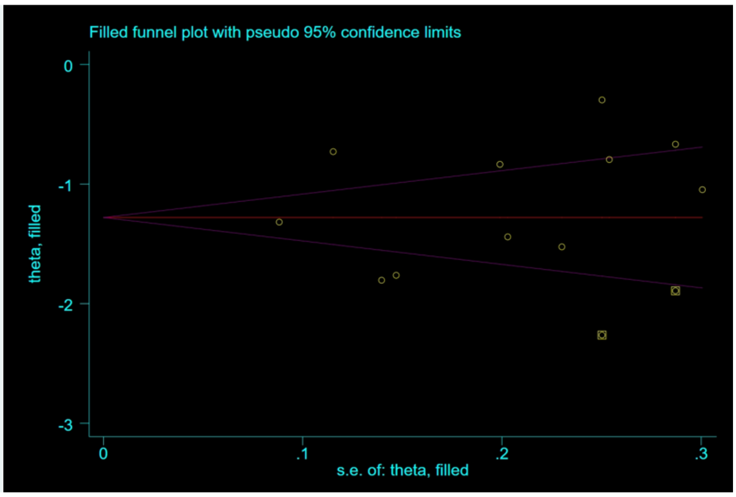

Supplementary Figure 38 Funnel Plot and Trim and Fill Plot of TC

Supplementary Figure 39

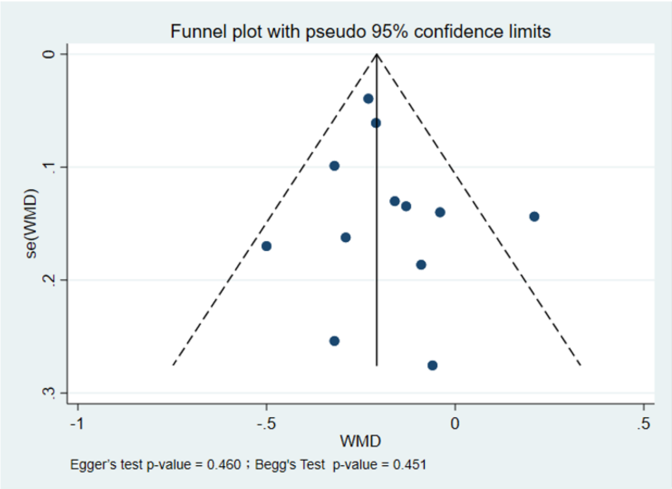

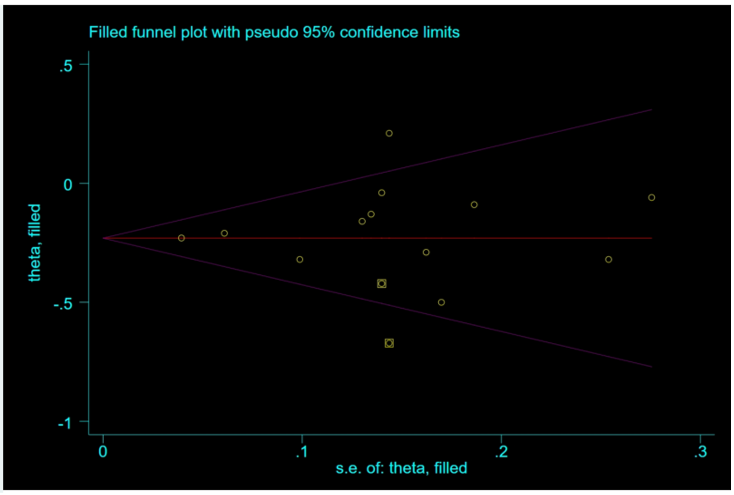

Supplementary Figure 39 Funnel Plot and Trim and Fill Plot of TG
